# Sources of airborne particulate matter-bound metals and spatial-seasonal variability of health risk potentials in four large cities, South Korea

**DOI:** 10.1007/s11356-021-18445-8

**Published:** 2022-01-06

**Authors:** Eunhwa Choi, Seung-Muk Yi, Young Su Lee, Hyeri Jo, Sung-Ok Baek, Jong-Bae Heo

**Affiliations:** 1 Institute of Construction and Environmental Engineering, Seoul National University, 1 Gwanak-ro, Gwanak-gu, Seoul, 08826 Republic of Korea; 2 Department of Environmental Health Sciences, Graduate School of Public Health, Seoul National University, 1 Gwanak-ro, Gwanak-gu, Seoul, 08826 Republic of Korea; 3 Department of Civil and Environmental Engineering, College of Engineering, Seoul National University, 1 Gwanak-ro, Gwanak-gu, Seoul, 08826 Republic of Korea; 4 Department of Environmental Engineering, Yeungnam University, Gyeongsan, 38541 Republic of Korea; 5 Busan Development Institute, 955 Jungangdae-ro, Busanjin-gu, Busan, 47210 Korea

**Keywords:** Health risk, Inhalation, Seasonal variability, Source apportionment, Spatial variability, PMF, Metal

## Abstract

Fifteen airborne particulate matter-bound metals were analyzed at 14 sites in four large cities (Seoul, Incheon, Busan, Daegu) in South Korea, between August 2013 and June 2017. Among the seven sources resolved by positive matrix factorization, soil dust and marine aerosol accounted for the largest and second largest portions in the three cities; however, in Seoul, soil dust and traffic occupied the largest and the second largest, respectively. Non-carcinogenic risk assessed by inhalation of eight metals (Cd, Co, Ni, Pb, As, Al, Mn, and V) was greater than the hazard index (HI) of 1 at four sites located at or near the industrial complexes. Cumulative incremental lifetime cancer risk (ILCR) due to exposure to five metals (Cd, Co, Ni, Pb, and As) exceeded the 10^−6^ cancer benchmark at 14 sites and 10^−5^ at six sites, which includes four sites with HI greater than 1. The largest contributor to ILCR was coal combustion in Seoul, Incheon, and Daegu, and industry sources in Busan. Moreover, industry sources were the largest contributors to non-carcinogenic risk in Seoul, Busan, and Daegu, and soil dust was in Incheon. Incheon had the highest HI in spring because of the higher contribution of soil dust sources than in other seasons. The higher ILCR in Incheon in spring and winter and higher ILCR and HI in Daegu in autumn were mainly due to the influence of industry or coal combustion sources. Statistically significant differences in the ILCR and HI values among the sampling sites in Busan and Daegu resulted from the higher contribution of industry sources at a certain site in the respective city.

**Supplementary Information:**

The online version contains supplementary material available at 10.1007/s11356-021-18445-8.

## Introduction

Exposure to particulate matter (PM) in the air has been linked to a range of adverse health outcomes, including respiratory infections, cardiovascular disease, and lung cancer (WHO 2006). Although the PM components that most impact health are still a matter of intense investigation, it is known that certain metals (or metalloids, hereinafter metals) are especially potent in causing DNA damage and cancer (WHO 2006), or lead to indirect damage by producing reactive oxygen and nitrogen species (Valko et al. 2005). PM has strong potential for adsorbing toxic metals and entering the human body mainly by inhalation, which can result in acute or chronic adverse health effects (Li et al. 2013).

Metals, including heavy metals, are naturally occurring elements found throughout the earth’s crust. Natural phenomena such as volcanic eruptions, weathering of rocks, soil erosion, sediment re-suspension, and metal evaporation from water have been reported to significantly contribute to metal pollution (Tchounwou et al. 2012; Briffa et al. 2020). However, the main cause of environmental pollution and human exposure to metals are due to anthropogenic activities such as mining, smelting and metal processing in foundries and refineries, coal burning in power plants, petroleum combustion, automobile operation, and roadworks (Tchounwou et al. 2012; Briffa et al. 2020). Environmental pollution by toxic metals has accelerated since the start of industrialization and urbanization. Given that metals released into living environments are not biodegraded and persist for a long time, chronic exposure to toxic metals in the living environment is a great threat to public health (Hu et al. 2012; Tchounwou et al. 2012; Liu et al. 2014; Lyu et al. 2017; Briffa et al. 2020). Nonetheless, the small contribution of metals to total suspended particles (TSP), PM_10_, or PM_2.5_ concentration resulted in considerably less attention for metal elements (Farahani et al. 2021).

Previous studies have resolved the emission sources of metals measured in living environments using multivariate models (Arruti et al. 2010; Du et al. 2019; Jiang et al. 2020; Fan et al. 2021). However, most past studies focused on capital cities or severely polluted industrial cities, and health risks due to inhalation of hazardous metals have rarely been assessed among cities or across multiple sites. Therefore, metal sources that influence carcinogenic and non-carcinogenic health risks in cities, seasonal and spatial variability of metal source contributions, and corresponding countermeasures are not adequately understood.

As South Korea is a country with a small area surrounded by the sea on three sides, its air quality is heavily influenced by marine aerosols from the sea, Asian yellow dust from the northwest, and anthropogenic air pollutants of foreign origins (Choi et al. 2010, 2013; Kim et al. 2020; Park et al. 2020, 2021). In addition, due to the high urbanization rate, 81% of the total population as of 2020 (World Bank 2018) and high population density (Statistics Korea 2019), there are many residential areas in South Korea that are close to industrial facilities and roads, and are thus affected by air pollutants from local anthropogenic sources. Thus, South Korea is an interesting area for studying the influence of various metal sources.

The aim of this study was to compare the exposure to TSP-bound metals among people at 14 sites spread across four metropolitan cities in South Korea, apportion metal sources using positive matrix factorization (PMF), and estimate the contribution of metal sources to both carcinogenic and non-carcinogenic health risks by inhalation of airborne metals. We also assess the spatial and seasonal variability of health risks to explore opportunities to reduce exposure to metals.

## Materials and methods

### Study site

The concentration of metals in the airborne particles was measured by season at 14 sites in the four largest cities in South Korea (In order of population: Seoul, Busan, Incheon, and Daegu), accounting for 35.3% of the total population of South Korea (Statistics Korea 2019). Seoul is the capital and largest metropolis of South Korea, with a population of 9.8 million as of 2017 (Statistics Korea 2019). Busan, South Korea’s second largest metropolis, is a coastal city located at the southeastern tip of the Korean Peninsula. Incheon is located in a coastal area close to Seoul, it is often enveloped in sea fog and affected by long-range transport of pollutants from industrial complexes in China, as well as Asian yellow dust formed through desertification (Choi et al. 2011, 2013). Daegu, the 4th largest city, is located inland with a basin topography surrounded by mountains.

**Fig. 1:**
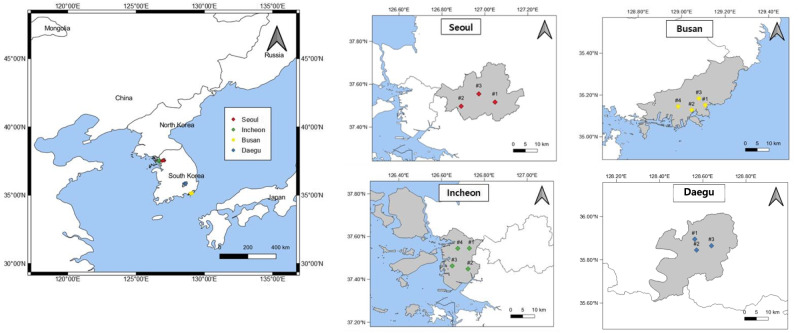
Study sites

Airborne particle samples were collected in three seasons (summer, autumn, and winter) at three sites in Seoul, and in four seasons at three to four sites in Incheon, Busan, and Daegu for more than seven consecutive days per season. The locations of each sampling site are shown in Fig. 1 and Table S1 in the Supporting Information (SI). Details about the sampling periods and the number of samples collected during the campaigns are presented in Table 1, and district classification (e.g., residential area, at or near industrial complexes, roadsides, port areas) of the sites are described in Table S1.

**Table 1:** Monitoring campaigns in four cities

Sampling sites	Number of samples^a^	Sampling period
**Seoul**		
- GN (Seoul #1)	30	Aug. 17 to 27, 2013 Nov.12 to 22, 2013 Feb.4 to 13, 2014
- GR (Seoul #2)	30	
- ST (Seoul #3)	25	
**Incheon**		
- GS (Incheon #1)	28	July 30 to Aug.5, 2014 Oct. 15 to 21, 2014 Jan.13 to 19, 2015 Mar.18 to 24, 2015
- GW (Incheon #2)	28	
- SE (Incheon #3)	28	
- YH (Incheon #4)	28	
**Busan**		
- GA (Busan #1)	39	Aug.18 to 27, 2015 Oct. 20 to 29, 2015 Jan. 19 to 28, 2016 Apr. 12 to 21, 2016
- SJ (Busan #2)	39	
- YS (Busan #3)	40	
- HJ (Busan #4)	40	
**Daegu**		
- NW (Daegu #1)	28	Nov.15 to 21, 2016 Feb. 10 to 16, 2017 Apr. 11 to 17, 2017 Jun. 21 to 27, 2017
- DM (Daegu #2)	28	
- MC (Daegu #3)	27	

^a^A total of 15 elements (Al, As, Ca, Cd, Co, Fe, K, Mg, Mn, Na, Ni, Pb, Ti, V, and Zn) were analyzed for each sample and however, some metals in the samples had missing values

### Metal measurement

TSP was collected using high-volume samplers (TEPNY1123, Tisch Environmental, USA) that operated for 24 h at a flow rate of 550–600 L/min. Quartz fiber filters (8 × 10″, QMA filter, Whatman, USA) were used for particle collection after pretreatment at approximately 400 °C for 4 h to remove any organic impurities. Pre- and post-field filters were conditioned in a room of constant humidity (45 ± 5%) and temperature (20 ± 1 °C) for 24 h and were gravimetrically tared following the USEPA IO-3.1 method. A total of 15 elements (Al, As, Ca, Cd, Co, Fe, K, Mg, Mn, Na, Ni, Pb, Ti, V, and Zn) were extracted with a solution of HCl (16.75%, v/v) and HNO_3_ (5.55%, v/v) using a microwave digester (Ethos, Milestone, Italy), and then quantified by inductively coupled plasma atomic emission spectrometry (ICP-AES; Optima 3000RL, Perkin Elmer, USA). Details for QA/QC, including analytical precision, method detection limits, and recovery rate, were described in Table S2 and a previous study (Kim et al. 2021).

The concentrations of PM_10_, NO_2_, and CO measured at the national monitoring stations where the TSP samples were collected or at the national monitoring station closest to the TSP sampling site were obtained from the Airkorea website (Korea Environmental Corporation 2021a). The mass concentrations of PM_10_ were measured by β-ray absorption method (BAM 1020, Met One Instruments, Inc., USA; FH 62 C14, Thermo Fisher Scientific Inc., USA; SPM-613 and PM-711, KIMOTO Electric Co., Ltd, Japan). Quality assurance and control (QA/QC) for PM_10_, NO_2,_ and CO analysis can be found in the relevant guidelines (Ministry of Environment of Korea and National Institute of Environmental Research 2021).

### Positive matrix factorization

Positive matrix factorization (PMF) is an effective tool for decomposing measured datasets into factor composition profiles and factor contributions (Paatero and Tapper 1994). The EPA PMF 5.0 was applied to the 15 metals measured at the 14 sites to identify emission sources and quantify the contributions of these sources to both carcinogenic and non-carcinogenic risks. The metal concentration data from sampling sites in the same city were bundled to obtain a PMF solution for each city. In addition, gaseous pollutants such as NO_2_ and CO were used as input concentration values for PMF analysis to help determine a specific source from the factor extracted by the PMF.

The following chemical mass balance equation is used to obtain factor contributions and profiles:

1 $${X}_{ij}=\sum\nolimits_{k=1}^{p}{G}_{ik}{F}_{kj}+{E}_{ij}$$

where ${X}_{ij}$ is a data matrix of metal *j* in sample *i*, *p* is the total number of sources, ${G}_{ik}$ is the contribution matrix of source *k* for sample *i*, ${F}_{kj}$ is the fraction of metal *j* for source *k*, and ${E}_{ij}$ is the residual for metal *j* in sample *i* and it can be acquired by minimizing the object function *Q*:

2 $$Q= \sum\nolimits_{i=1}^{n}\sum\nolimits_{j=1}^{m}{\left[\frac{{X}_{ij}-{\sum\nolimits }_{k=1}^{p}{G}_{ik}{F}_{kj}}{{u}_{ij}}\right]}^{2}$$

where ${u}_{ij}$ represents the uncertainty of metal *j* in sample *i*, *n* is the number of samples, and *m* is the number of species.

Because individual data points can be weighted by reported uncertainties (Paatero et al. 2014), the PMF model permits it to handle below the detection limit or missing data and generate more physically explainable factors (Paatero et al. 2014; Almeida et al. 2020). Hence, PMF has been widely applied to quantify the contributing sources of PM_2.5_ (Heo et al. 2009), NMVOCs (Choi et al. 2010, 2011), or PM constituents such as polycyclic aromatic hydrocarbons and metal elements (Jiang et al. 2020; Fan et al. 2021; Farahani et al. 2021; Kim et al. 2021).

As a result, 15 metal species from 85 samples in Seoul, 112 samples in Incheon, 158 samples in Busan, and 83 samples in Daegu were included in the PMF model for analysis (Table 1; Table S3).

### Exposure and health risk assessment

The exposure concentration (EC) of metals via inhalation was calculated using the following equation (US EPA 2009):

3 $${EC}_{inh}=C\times \frac{ET\times EF\times ED}{AT}$$

where *EC*_*inh*_ is the exposure concentration through inhalation averaged over a lifetime (µg/m^3^); *C* is the concentration of metal at the sampling site (µg/m^3^); *ET* is the exposure time (hours/day), which was taken as 6 h day^−1^; *EF* is the exposure frequency (days/year), assumed to be 350 days year^−1^; ED is the exposure duration (year), which was taken as expected life expectancy after adulthood in Korea, 63.7 years; and *AT* is the averaging time (hours), which was assumed to be ED × 365 × 24.

Carcinogenic risk is estimated as the incremental probability of developing cancer over a lifetime as a result of exposure to a potential carcinogen (US EPA 1989):

4 $$ILCR=({EC}_{inh}\times IUR)$$

where *ILCR* is a unitless incremental lifetime cancer risk and *IUR* is the inhalation unit risk (µg/m^3^) ^−1^.

The human non-carcinogenic effect for the *i*th metal is defined as follows:

5 $${HQ}_{i}={EC}_{i inh}/{RfC}_{i}$$

6 $${HQ}_{is}={EC}_{is inh}/{RfC}_{i}$$

where *HQ*_*i*_ is the hazard quotient for the *i*th metal, unitless; *RfC*_*i*_ is the chronic inhalation reference concentration for the airborne *i*th metal (µg/m^3^); *HQ*_*is*_ is the hazard quotient for the *i*th metal in source *s*; and *EC*_*is* inh_ is the exposure concentration to the *i*th metals in source *s* (µg/m^3^).

The hazard index (*HI*) for chronic exposure to multiple toxic metals is calculated using the following equations:

7 $$HI=\sum\nolimits_{i=1}^{n}{HQ}_{i}$$

8 $${HI}_{s}=\sum\nolimits_{i=1}^{n}{HQ}_{is}$$

9 $${HI}_{s, \mathrm{total}}=\sum\nolimits_{s=1}^{p}{HI}_{s}$$

where *n* is the number of metals, *HI* is the *HI* at each study site or city for chronic exposure to metal 1 through *n*, unitless, *HI*_*s*_ is the *HI* in source *S*, and *HI*_*s*,total_ is the HI obtained by sum of the *HI*_*s*_ from all sources.

For a deterministic derivation of the inhalation intake of metals, the arithmetic means of the metal concentrations measured at 14 sites were used as *C* in Eq. (3) (Table S3). To estimate the inhalation intakes of metals by source, the fraction of each metal in the sources resolved by PMF and the source contributions were used as input data, *C* in Eq. (3). In this study, *IUR* (µg/m^3^) ^−1^ and *RfC* (µg/m^3^) were obtained from the USEPA’s Integrated Risk Information System (IRIS) (US EPA 2021). If two or more toxicity values differed for any metal, the values listed in the IRIS generic tables were prioritized (Tables 2 and 3). Metals for which no *RfC* and no *IUR* values were available were excluded from the risk estimation. As a result, five metals (Cd, Co, Ni, Pb, and As) and eight metals (Cd, Co, Ni, Pb, As, Al, Mn, and V) were included in the calculation of carcinogenic and non-carcinogenic risks, respectively.

**Table 2:** Toxicological data and carcinogenic risk

**Chemical**	**Unit risk** (µg/m^3^)^−1^	**Unit risk source** ^a^	**Unit risk basis**	**Chemical-specific risk**^b^			
				**Seoul** (*N* = 85)	**Incheon** (*N* = 112)	**Busan** (*N* = 128, 158)^c^	**Daegu** (*N* = 83)
**As**	0.0043	IRIS	Air	4.21E-06 (62.9)	6.56E-06 (67.1)	3.22E-06 (41.9)	4.82E-06 (39.8)
**Co**	0.009	PPRTV	Air	1.62E-06 (24.2)	2.03E-06 (20.8)	2.12E-06 (27.6)	5.69E-06 (47.0)
**Ni**	0.00024	IRIS	Air	2.62E-07 (3.9)	5.14E-07 (5.3)	1.14E-06 (14.8)	7.52E-07 (6.2)
**Cd**	0.0018	IRIS	Air	5.11E-07 (7.6)	5.27E-07 (5.4)	1.07E-06 (13.9)	6.82E-07 (5.6)
**Pb**	0.000012	OEHHA	Air	9.71E-08 (1.4)	1.39E-07 (1.4)	1.35E-07 (1.8)	1.60E-07 (1.3)
**Incremental lifetime cancer risk**		By the sum of chemical-specific risks using measured data		6.70E-06 (100)	9.77E-06 (100)	7.68E-06 (100)	1.21E-05 (100)
		By the sum of source specific risks using PMF results		6.69E-06	1.03E-05	7.59E-06	1.22E-05

^a^(US EPA 2021)

^b^The numbers in parentheses are the proportion (%) of chemical-specific cancer risk in cumulative ILCR in the city

^c^*N* = 128 for Pb; *N* = 158 for other metals

**Table 3:** Toxicological data and non-carcinogenic risk

**Chemical**	**RfC** (µg/m^3^)	**RfC source**	**RfC basis**	**Hazard quotient**^c^ (HQ_i_)			
				**Seoul** (N = 85)	**Incheon** (N = 112)	**Busan** (N = 128, 156, 158)^d^	**Daegu** (N = 83)
**As**	0.015	OEHHA^a^	Air	6.53E-02 (12.4)	1.02E-01 (11.5)	4.99E-02 (4.4)	7.47E-02 (8.8)
**Co**	0.006	PPRTV^a^	Air	3.00E-02 (5.7)	3.76E-02 (4.3)	3.93E-02 (3.5)	1.05E-01 (12.4)
**Ni**	0.014	Cal EPA^a^	Air	7.79E-02 (14.8)	1.53E-01 (17.3)	3.40E-01 (29.7)	2.24E-01 (26.3)
**Cd**	0.01	ATSDR^a^	Air	2.84E-02 (5.4)	2.93E-02 (3.4)	5.93E-02 (5.2)	3.79E-02 (4.5)
**Pb**	0.15	USEPA NAAQS^b^	Air	5.40E-02 (10.2)	7.70E-02 (8.8)	7.47E-02 (6.6)	8.91E-02 (10.5)
**Al**	5	PPRTV^a^	Air	6.38E-02 (12.1)	1.27E-01 (14.5)	5.56E-02 (4.9)	6.35E-02 (7.5)
**V**	0.1	ATSDR^a^	Air	1.01E-02 (2.0)	2.56E-02 (2.9)	1.88E-02 (1.7)	1.03E-02 (1.3)
**Mn**	0.05	IRIS^a^	Air	2.00E-01 (37.8)	3.34E-01 (37.7)	5.09E-01 (44.4)	2.48E-01 (29.1)
**Hazard index**		By the sum of chemical-specific risks using measured data (HI)		5.29E-01 (100)	8.85E-01 (100)	1.15 (100)	8.53E-01 (100)
		By the sum of source-specific risks using PMF results (HI_s, total_)		5.11E-01	9.26E-01	1.11	8.24E-01

^a^(US EPA 2021)

^b^(State of Michigan, USA 2016)

^c^The numbers in parentheses are the proportion (%) of HQ_i_ in HI in the city

^d^*N* = 128 for Pb; *N* = 156 for Al; *N* = 158 for other metals

The pathway benchmark cancer risk value, for example, one-in-a-million, one-in-a hundred thousand, or one-in-ten thousand, refers to the increased chance of developing cancer over a lifetime as a direct result of breathing air containing the chemical (US EPA 1989). The pathway hazard index (*HI*) was determined by adding all *HQ*_*i*_ values for the metals through the inhalation route at the same site (Eq. (7); Table 3). The HI for each source (*HI*_*s*_) was also determined by adding all the *HQ*_*i*_ values for eight metals within the same source (Eq. (8)), and the pathway HI by PMF (*HI*_*s*,total_) was estimated by adding all of the *HI* values of every source (*HI*_*s*_) at the same location (Eq. (9); Table 3). A total HI (i.e., *HI* or *HI*_*s*,total_) greater than 1 indicates the potential for adverse health effects (US EPA 2009).

### Statistical analysis

Data analysis was performed using the Statistical Package for Social Sciences (version 21; IBM Corp., Armonk, NY, USA). The strength and direction of the monotonic relationships between estimated health risks (i.e., ILCR and HI) and the mass concentrations of TSP, PM_10_, and metals, and between the estimated health risks and the contributions of metal sources extracted by PMF were measured by applying Pearson’s correlation coefficient. One-way ANOVA with Tukey’s significant difference test was used to determine whether there were any statistically significant differences between seasonal means of TSP, PM_10_, HI, ILCR, and metal source contributions in a city, and between site-specific means within a city.

## Results and discussions

### Spatial distribution of measured metals

**Fig. 2:**
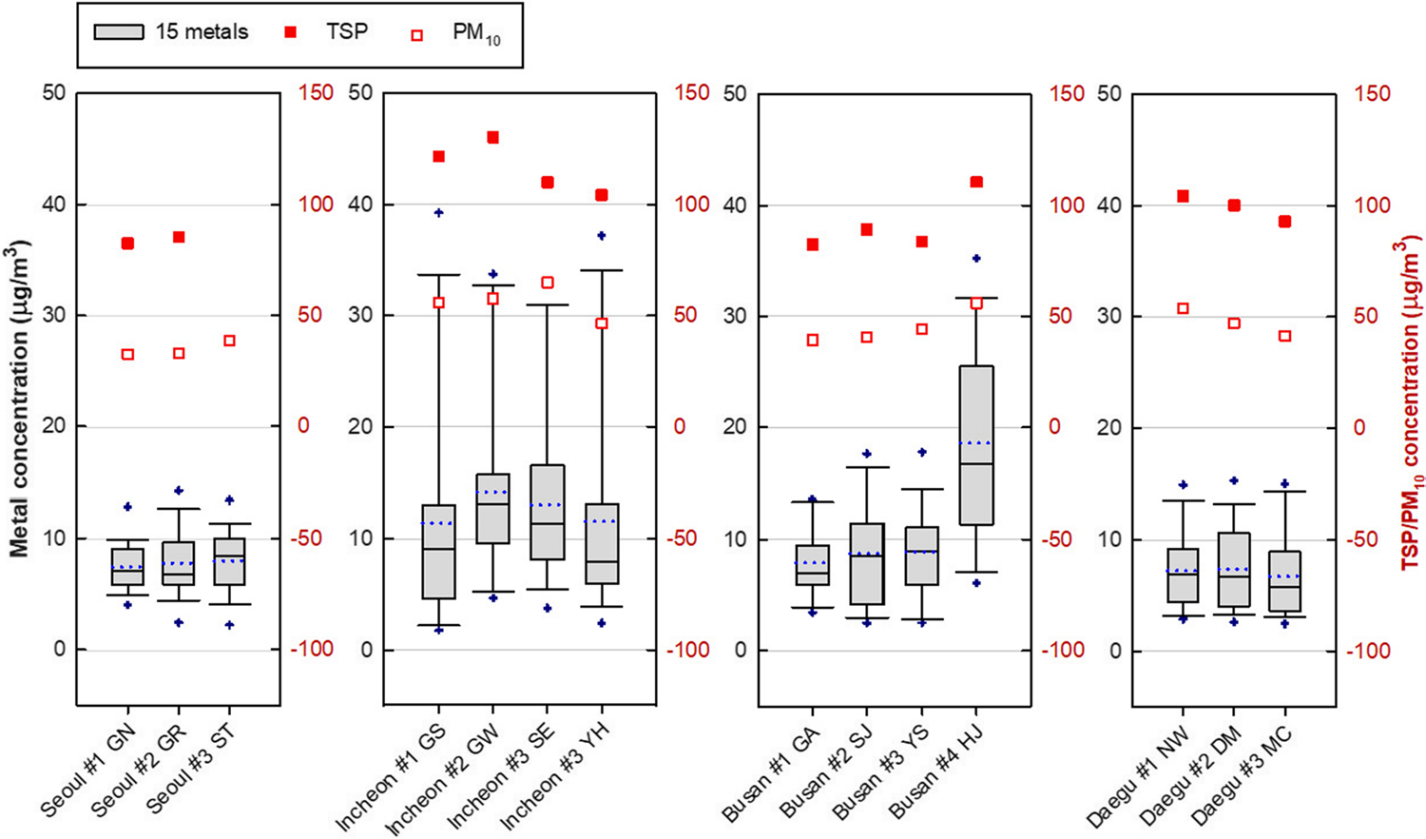
Mass concentrations of 15 metals, TSP, and PM_10_ by site during sampling period

Mean concentrations of TSP and PM_10_ were the highest in Incheon (117.26 µg/m^3^, 56.26 µg/m^3^) and lowest in Seoul (83.87 µg/m^3^, 34.74 µg/m^3^) during the measurement period (Fig. 2; Table S3). The average mass concentration of the 15 metals was the highest in Incheon at 12.8 µg/m^3^, followed by Busan (10.5 µg/m^3^), Seoul (7.7 µg/m^3^), and Daegu (7.1 µg/m^3^) (Fig. 2). Meanwhile, Busan #4 HJ site had the highest average mass concentration (17.3 µg/m^3^) of the 15 metals among the 14 sites, which resulted in a large variation in the metal mass concentration in Busan.

The most abundant elements at 14 sites were Al, Fe, and Ca, which rank 3rd, 4th, and 5th in terms of abundance of elements in the earth’s crust, respectively (Mason et al. 2021). Ca is a common element in seawater and the earth’s crust, and similarly, the mass concentrations of Na, Mg, and K that are abundant in seawater and the earth’s crust showed high concentrations at all 14 sampling sites (Enghag 2008; Table S3).

As for the five metals used in both ILCR and HI estimations (Cd, Co, Ni, Pb, and As), the highest and second highest concentrations of Cd, Co, Ni, and Pb were found at Busan #4 HJ and Daegu #2 DM sites located at and near industrial complexes, respectively (Table S3). As a result, there were large variations in the concentrations of Co, Ni, and Pb between the measurement sites in Daegu, and in the concentrations of Co, Ni, Pb, and Cd between the measurement sites in Busan. On the other hand, among the four cities, the concentrations of Pb and As were higher and uniformly distributed in Incheon, the city closest to large and small-scale coal-using plants and power plants compared to those in other cities (Korea Environmental Corporation 2021b).

As for the three metals used in the HI estimation (Al, Mn, and V), the mass concentration of Al, which is the third most abundant component of the earth’s crust, was higher in Incheon than the other three cities. While Mn, the 12^th^ most abundant component of the earth’s crust, was also the highest in Incheon, the Mn concentration at Busan #4 HJ was the highest among the 14 sampling sites. The concentration of V, which is emitted from residual oil combustion, was the highest in Incheon. Among the 14 sites, it was the highest at Incheon #3 SE and second highest at Busan #2 SJ, both of which are very close to the port, and can be classified as a port area (Fig. 1; Table S1).

### Source identification and apportionment using PMF

**Fig. 3:**
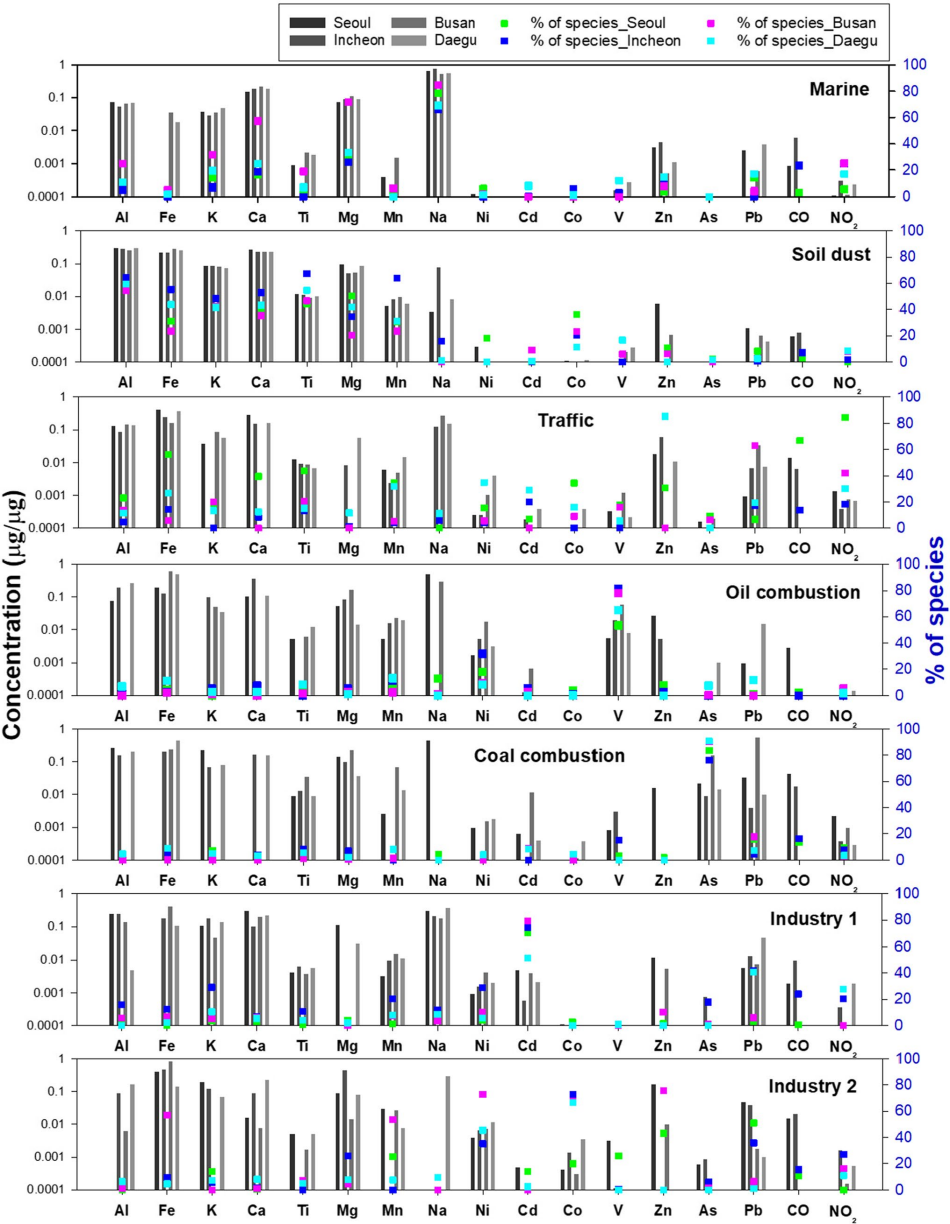
Source profiles of factors extracted by PMF

**Fig. 4:**
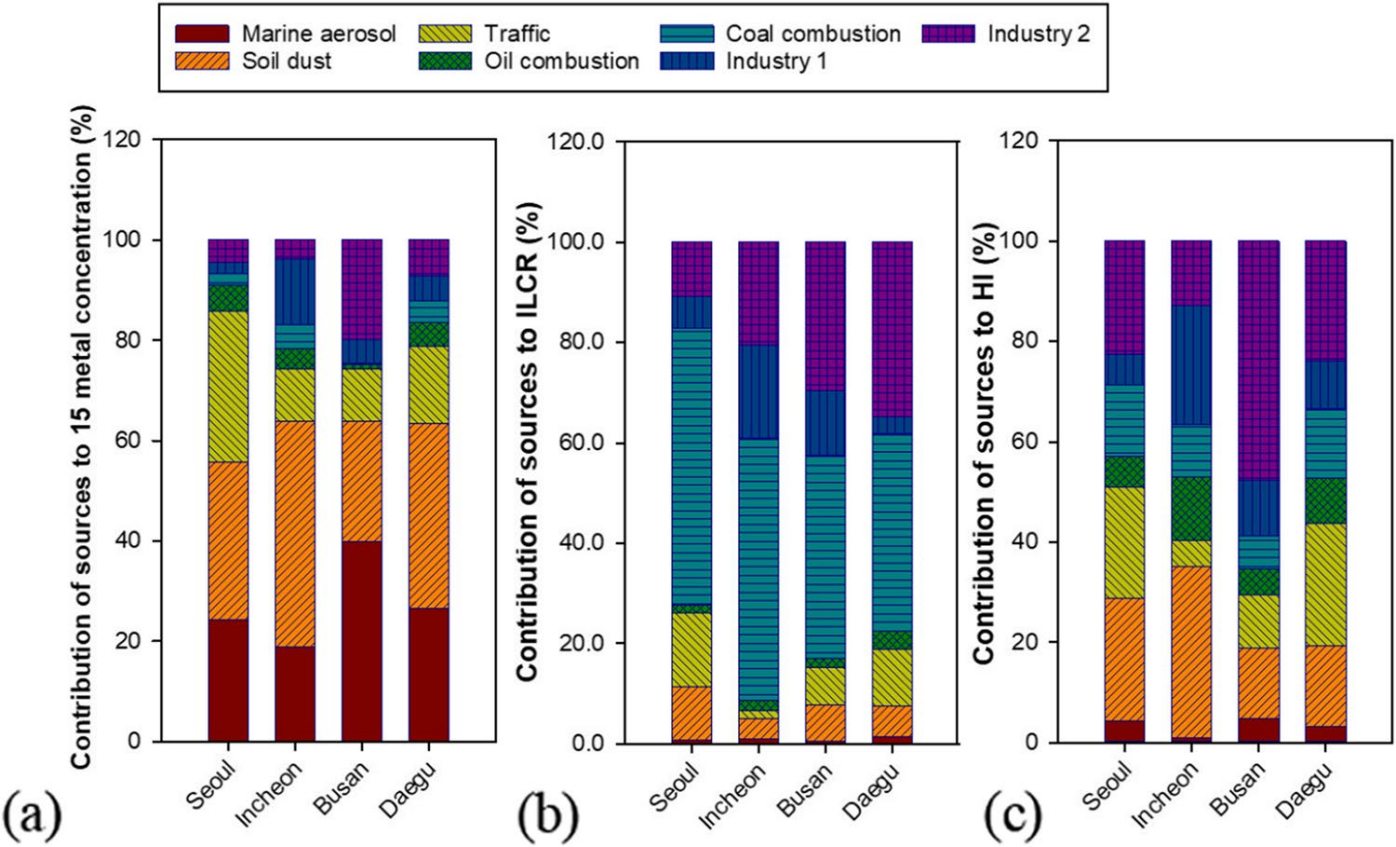
Contribution (%) of sources to (a) metal concentration, (b) ILCR, and (c) HI

This study considered four to eight factors for optimal PMF results for each city. The number of factors was determined when sources with well-known source profiles (i.e., soil dust, marine aerosol, traffic, and oil combustion) did not mix with each other, and when these sources were not split into two. The most appropriate factor solutions were chosen based on the minimum Q value. The solutions were assessed according to the distributions of the scaled residuals, correlations between the source contributions (Paatero et al. 2005), and displacement analysis (US EPA 2014). In the four cities, the most meaningful solution was provided by seven factors: marine aerosol, soil dust, traffic-related, oil combustion, coal combustion, and two industrial activity-related sources (Figs. 3 and 4; Table S4). The *R*^2^ values for the linear regression between the measured and predicted total metal mass concentrations were 98.6% in Seoul, 99.1% in Incheon, 92.8% in Busan, and 99.7% in Daegu.

The marine aerosol sources were composed of Na, Ca, Mg, K, and Al (Enghag 2008), and accounted for the largest portion of 15 metal concentrations in Busan (39.8%) (Fig. 4).

The soil sources were dominated by typical crustal components such as Al, Fe, Ca, Mg, K, and Ti (Vouk and Piver 1983). Co is a tracer of soil dust (Vouk and Piver 1983) and is present in road dust (Zannoni et al. 2016). However, the concentration of Co in this soil source was found to be as high as that in traffic-related sources in Seoul, Incheon, and Busan, suggesting that the soil dust was mixed with road dust to some extent. In addition, the higher contribution of the soil source on weekdays than on weekends in all four cities supported the mixing of soil dust with road dust. Soil dust sources were the largest source contributing to 15 metal concentrations in Seoul, Incheon, and Daegu, accounting for 31.7%, 45.0%, and 36.7%, respectively.

The third source, traffic-related sources, including both exhaust and non-exhaust emissions, contained high concentrations of Fe, Zn, Pb, Mn, Ti, CO, and NO_2_ (Liu et al. 2014; Zannoni et al. 2016). Brake pads and tire wear are known sources of Mn, Zn, Fe, and Ti (Apeagyei et al. 2011; Zannoni et al. 2016). The presence of Co, Ti, and Pb is attributed to the tear of the road paint (Yu et al. 2016; Zannoni et al. 2016). This source showed a higher contribution during weekdays compared to weekends in Seoul, Busan, and Daegu.

The oil combustion source was indicated by high contributions of V and Ni, which are elements characteristic of residual oil combustion (Vouk and Piver 1983).

The coal combustion source was characterized by high loadings of As and Pb (Vouk and Piver 1983; Tian et al. 2014; Yu et al. 2016).

The sixth and seventh profiles are related to industrial activities. The sixth profile, assigned to industry I, was characterized by a high contribution of Cd, with dominant sources of air emissions being waste incineration and non-ferrous metal production. (Nordic Council of Ministers 2003). The seventh profile was assigned to industry II. The proportion of Ni in this source was higher than that in other sources (Fig. 3). However, the profile contained different dominant metals in each city. The industry II source was characterized by a strong loading of Zn, Pb, V, Mn, Cd, and Co in Seoul, by Co, Pb, Mg, NO_2_, and CO in Incheon, by Zn, Co, Fe, Mn, and NO_2_ in Busan, and by Co in Daegu. Industrial activity-related two sources showed a higher contribution during weekdays than on weekends in all four cities.

As shown in Fig. 4(a), the average proportion of soil dust and marine aerosols accounted for more than 50% of the 15 metal concentrations (55.9% in Seoul and from 63.4 to 63.9% in Incheon, Busan, and Daegu). Two industrial activity-related sources accounted for the highest proportion in Busan (24.6%), followed by Incheon (16.9%), Daegu (12.1%), and Seoul (6.8%). The contribution of coal combustion to 15 metal concentrations was the highest at 4.6% in Incheon, followed by Daegu (4.3%), Seoul (2.1%), and Busan (0.18%), which is in line with proximity to coal power plants or power plants partially using coal (Korea Environmental Corporation 2021b).

The PMF results described the characteristics of the four cities through source contributions and source profiles of 15 metals (Figs. 3 and 4(a); Table S4). Traffic sources (30.0%) in Seoul, the capital of South Korea, accounted for a larger proportion than in other cities. The higher proportion of soil dust sources (45.0%) in Incheon was in line with the findings regarding the influence of Asian yellow dust reported in previous studies (Choi et al. 2013; Kim et al. 2020; Park et al. 2021). A high concentration of Asian yellow dust was observed in Incheon during the measurement period (i.e., March 20 to 22, 2014). The mean concentration of 15 metals during this Asian yellow dust event at four sampling sites in Incheon was 34.7 µg/m^3^ corresponding to more than three times mean 15-metal concentration (10.0 µg/m^3^) during normal days. The PM_10_ concentration during the same Asian dust event was in line with the 15-metal concentration, recording an average of 132.3 µg/m^3^ at the four sampling sites. There was no sampling in Seoul during spring in this study; however, many studies have reported an increase in PM_2.5_ or PM_10_ in Seoul during the spring season or Asian dust events in spring (Heo et al. 2009; Kim et al. 2020; Park et al. 2020, 2021). Meanwhile, Busan, a coastal city located at the southeastern tip of South Korea, had the highest contribution of marine aerosols (39.8%) to the total metal concentration among the four cities (Fig. 4; Table S4). At Daegu #1 NW, adjacent to an expressway and an express bus terminal, the contribution of traffic sources was much higher (31.8%) than at the other two sites in the city (Table S4). Because the Daegu #1 NW site is situated at a school in an industrial complex, there is a lot of traffic of cargo vehicles as well as express buses, and there are many vehicle repairs. In the traffic source in Daegu, metals such as Ni and Co were higher than those in other cities (Fig. 3).

There were previous studies on source apportionment of PM_2.5_ mass concentrations measured in Seoul, Incheon #3 SE, and Busan #3 YS (Choi et al. 2013; Jeong et al. 2017; Park et al. 2020). Because only TSP-bound metals were included in source apportionment in this study, secondary inorganic sources that accounted for the largest portion of PM_2.5_ mass concentration were not extracted in this study (Table S4). While traffic sources constituted the second largest portion of PM_2.5_ mass concentration in Seoul, Incheon #3 SE, and Busan #3 YS city/sites in previous studies, traffic sources were the second, the fourth, and the third largest source of TSP-bound metal mass concentrations in Seoul, Incheon # 3 SE, and Busan # 3 YS respectively. On the other hand, the contributions of marine aerosol and soil dust sources to TSP-bound metal concentration noticeably increased compared to the contribution of them to the PM_2.5_ mass concentrations.

### Risk assessment

**Fig. 5:**
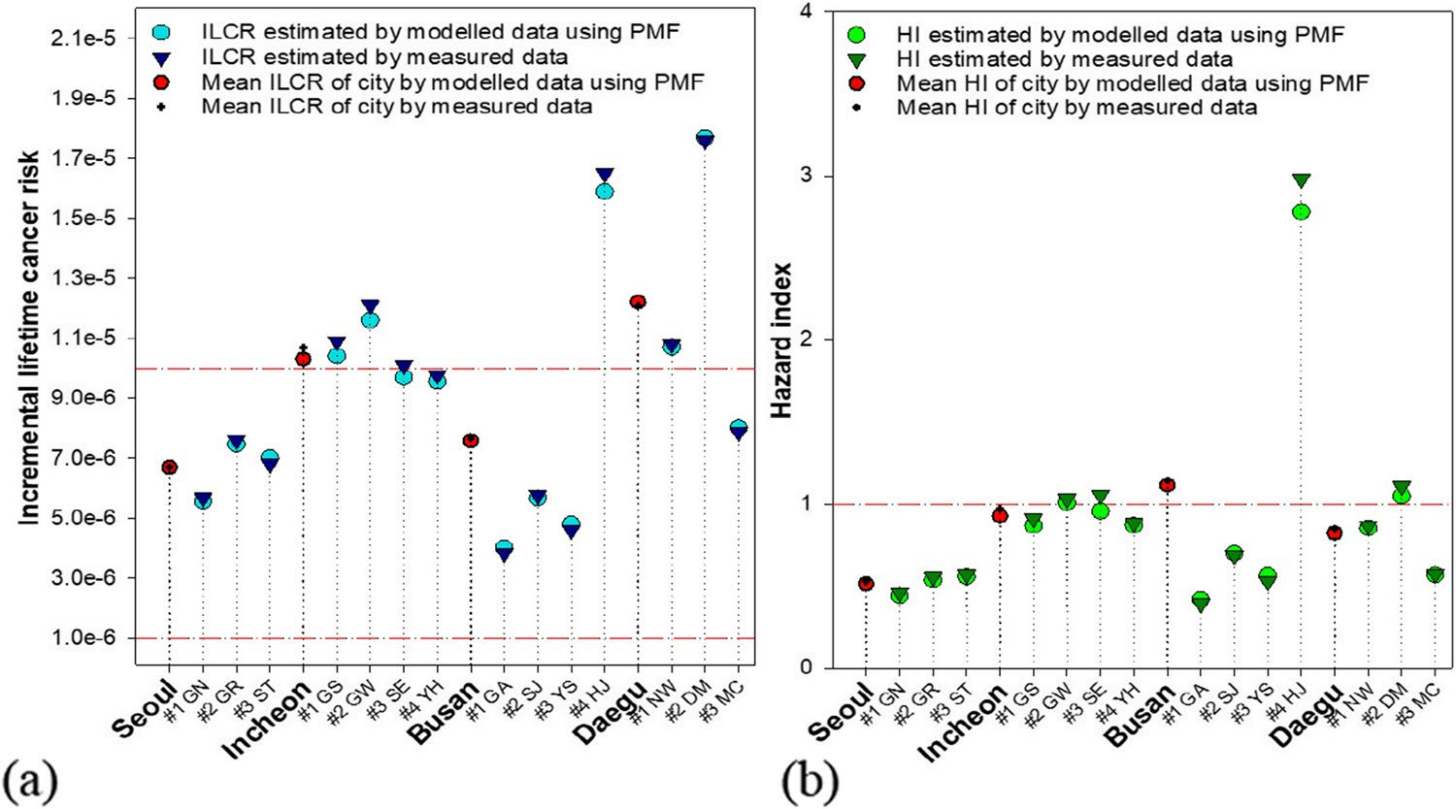
ILCR and HI values estimated at 14 sites in four cities using modelled data and measured data: (a) ILCR and (b) HI

The carcinogenic and non-carcinogenic risks estimated using the measured data are shown in Tables 2 and 3 and Fig. 5. The non-carcinogenic risk due to exposure to eight metals was greater than the HI of 1 at four sites located in or near the industrial complexes (Fig. 5; Table S1). Cumulative carcinogenic risks estimated using arithmetic mean concentrations of the five metals were greater than the one-in-a-million benchmark levels at all 14 sites and one-in-a hundred thousand at six sites, including four sites with HI greater than 1 (Fig. 5). Meanwhile, there were differences in estimated ILCR/HI values between measured values and reconstructed values by PMF. The average differences in the health risk values calculated by two data sets at 14 sites were 2.8% for ILCR and 4.2% for HI. To examine the characteristics of sources affecting carcinogenic and non-carcinogenic risks, the results of risk assessment using the reconstructed contribution by the PMF are presented hereinafter.

### Estimated carcinogenic and non-carcinogenic risks

**Fig. 6:**
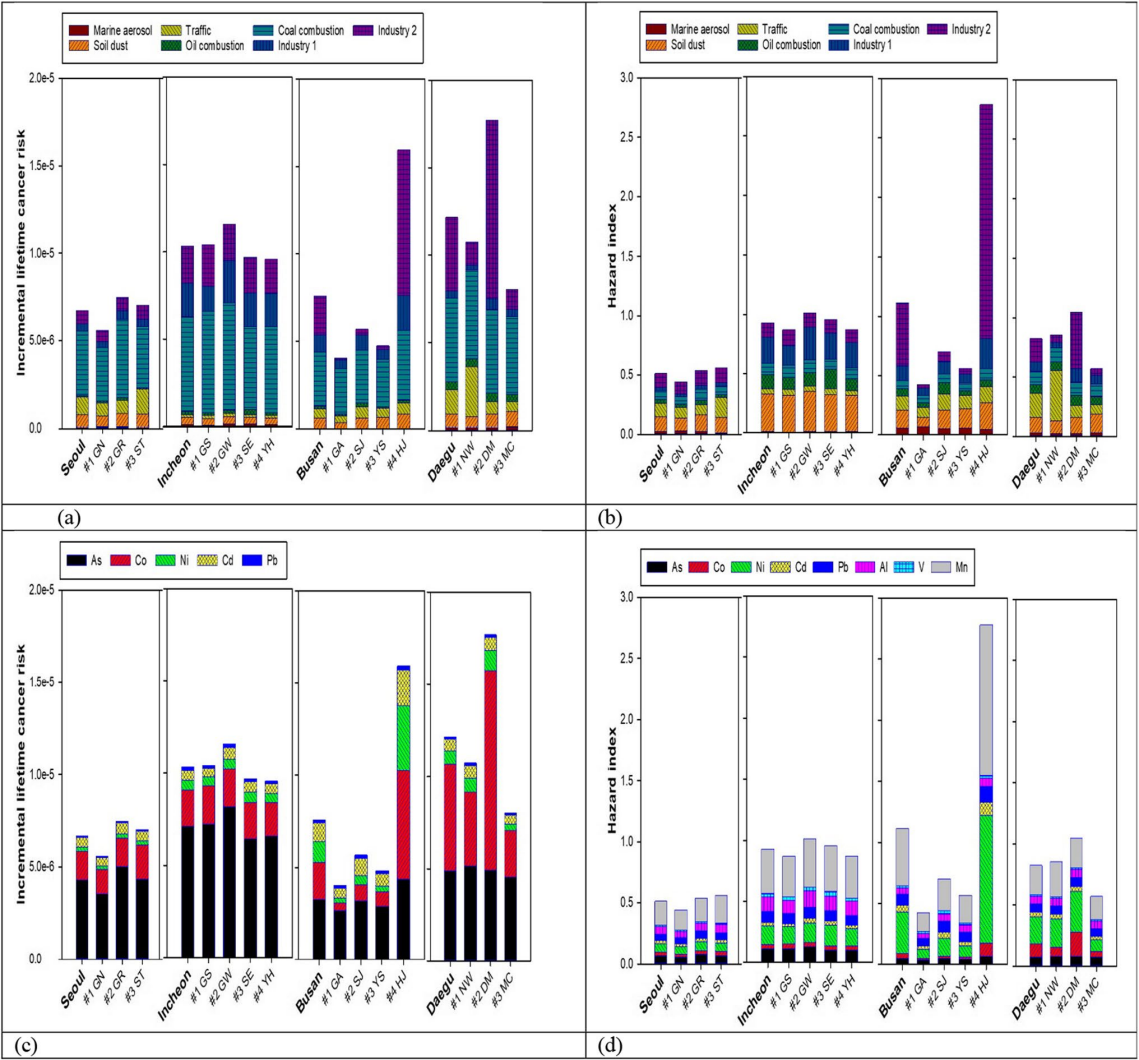
ILCR and HI values shown as contribution of sources and chemicals (a) contribution of sources to ILCR; (b) contribution of sources to HI; (c) contribution of chemicals to ILCR; (d) contribution of chemicals to HI

There was a maximum difference of 4.4 times between the lowest ILCR, at Busan #1 GA (4.01E-06), and the highest ILCR, at Daegu #2 DM (1.77E-05) (Fig. 5 and 6; Table S5). The ILCR estimated at the three measurement sites in Seoul was relatively low and even between 5.57E-06 and 7.48E-06. Similarly, ILCRs estimated at the four sites in Incheon were distributed evenly; however, the average ILCR in Incheon exceeded the 10^−5^ benchmark level. ILCRs estimated at three sites in Busan were between 4.01E-06 and 5.68E-06, much lower comparison to the Busan #4 HJ site (1.59E-05), which is located at a school in an industrial complex. The ILCRs in Daegu showed a relatively large variation among the three measurement sites.

The estimated non-carcinogenic risks associated with the eight metals in the atmosphere at three sites in Seoul ranged from 0.44 to 0.56 (Fig. 5; Table S6). The estimated HI at the Busan #4 HJ site was the highest among the 14 sites (2.78), resulting in an average HI value of 1.11 in Busan, despite the HI values (0.42, 0.70, and 0.56) at the other three sites in Busan being much lower than 1. The mean HI values in Incheon and Daegu were smaller than 1, but the HI values at one site in Incheon (1.01 at Incheon #2 GW) and Daegu (1.05 at Daegu #2 DM) were greater than 1.

According to the ANOVA test, the ILCR and HI values estimated at sampling sites in Seoul and Incheon were not statistically different. However, the ILCR and HI values at Busan #4 HJ were significantly different from those at the other three sites (*p* < 0.001) in Busan. Similarly, the ILCR in Daegu #2 DM was significantly different from those at Daegu #1 NW (*p* < 0.05) and Daegu #3 MC (*p* < 0.01), and HI at Daegu #2 DM was significantly different from that at Daegu #3 MC (*p* < 0.01). These variabilities were mainly due to the different contributions of industry II sources at the Busan #4 HJ and Daegu #2 DM sites (Fig. 6).

### Toxic metals and sources contributing to carcinogenic and non-carcinogenic risks

The metals contributing the most to carcinogenic risks were As in Seoul, Incheon, and Busan and Co in Daegu, as presented in Table 2 and Fig. 6. The largest contributors to carcinogenic risk were coal combustion in three cities: Seoul (55.1%), Incheon (52.4%), and Daegu (39.4%), and industrial activity-related sources in Busan (42.4%) (Fig. 4; Table S5). A similar result was found in a previous study conducted in Tianjin, China (Tian et al. 2021). Industrial emissions (34%) and coal combustion (31%) were the largest contributors to the cancer risk of atmospheric heavy metals during the warm and cold seasons, respectively. Contributions of coal combustion sources to carcinogenic risks (39.4% to 55.1%) in the cities were remarkably enhanced compared to contributions to metal mass concentration (0.18% to 4.64%). Daegu had the lowest average 15 metal concentration (Table S3); however, the ILCR value was the highest among the four cities due to the second highest contribution of coal combustion (4.25%) and the high proportion of Co from sources related to industrial activity and traffic (Fig. 3). Due to the highest concentration of As and the highest contribution of coal combustion source (4.64%) to 15 metal concentration, Incheon had the second highest ILCR value among the four cities. Seoul, which had the lowest contribution of industrial activity-related sources to 15 metal concentrations, showed the lowest carcinogenic and non-carcinogenic health risks among the four cities**.**

According to the British Petroleum Company plc (BP), in 2020, South Korea’s coal consumption ranked 8th in the world after China, India, the United States, Japan, South Africa, Russia, and Indonesia (BP 2021). To date, the co-benefit removal effects of conventional PM, sulfur dioxide, and nitrogen oxide control devices have gradually lowered the emissions of toxic elements, including As, Pb, Ni, Cd, Cr, and Hg (Li et al. 2013). However, coal consumption in the Asia–Pacific region continues to increase (Koplitz et al. 2017; BP 2021) and coal-fired power plants and coal combustion are still regarded as among the most important contributors to anthropogenic hazardous trace elements (Sb, As, Cr, Pb, Cd, Hg, Ni, Se, Be, Mn, and Co) pollution (Pandey et al. 2011; Tian et al. 2014). Hence, the need for advanced technologies and integrated management strategies to control hazardous elements has been emphasized in previous studies (Li et al. 2013; Tian et al. 2014).

Mn was the largest contributor to non-carcinogenic risk, and Ni was the second largest contributor, with the two metals accounting for more than 50% of the non-carcinogenic risk in all four cities (Table 3 and Fig. 6). In addition, higher contributions of As in Incheon and Daegu, Ni and Co at Busan #4 HJ, Daegu #1 NW, and Daegu #2 DM sites, and Al in Incheon to non-carcinogenic risks were characteristically observed (Fig. 6). Busan showed the highest HI value (1.11) of the four cities due to Mn, Ni, Co, and Cd, mainly emitted from industrial sources at the Busan #4 HJ site (Table 3; Figs. 5 and 6; Table S6). Risk analysis coupled with PMF results showed that soil dust was the biggest contributor to non-carcinogenic risk in Incheon (34.1%), located in northwest Korea (Table S6). Combining the proportions of the two industrial activity-related sources, they accounted for the largest proportion of non-carcinogenic risk in three cities (28.6% in Seoul, 58.8% in Busan, and 33.4% in Daegu). Thus, the contributions of industrial activity-related sources to non-carcinogenic risks increased noticeably from the contributions (6.8% in Seoul, 24.6% in Busan, and 12.1% in Daegu) to 15 metal concentrations (Fig. 4; Tables S4 and S6).

The importance of managing anthropogenic sources, such as coal combustion and industrial sources, was identified by the higher Pearson correlation coefficients between the estimated health risks and source contribution (Table S7). Strongly correlated sources with ILCR (i.e., Pearson’s *r*, greater than 0.7; *p* < 0.001) in the four cities were coal combustion or industry sources. Similarly, sources strongly correlated with HI in the four cities were industry sources (Pearson’s *r*, greater than 0.7; *p* < 0.001). Additionally, in Incheon, soil dust (Pearson’s *r*, 0.858; *p* < 0.001) and oil combustion (Pearson’s *r*, 0.739; *p* < 0.001) sources were strongly correlated with HI.

Meanwhile, the HI values were strongly correlated with mass concentrations of TSP, PM_10_, 15 metals, and five metals (Pearson’s *r* on average at 14 sites, 0.767, 0.781, 0.779, and 0.760, respectively; *p* < 0.01) at 14 sites, except for PM_10_ mass concentration at one site, Busan #1 GA (Pearson’s *r*, 0.267; *p* > 0.05) (Table S8). Similarly, ILCR was strongly correlated with the mass concentration of the five metals (Pearson’s *r* on average at 14 sites, 0.738; *p* < 0.01). However, ILCR was weakly correlated with TSP (Pearson’s *r* on average at 13 sites, 0.483) and 15 metals (Pearson’s *r* on average at 14 sites, 0.369) and moderately correlated with PM_10_ (Pearson’s *r* on average at 14 sites, 0.501). The correlations between ILCR and mass concentrations of TSP, PM_10,_ and 15 metals were not statistically significant at three, three, and four sites, respectively, among the 14 sites. It implies that the mass concentrations of TSP and PM_10_ may not be appropriate for managing carcinogenic risks caused by airborne metals, given that the Cd, Co, Ni, Pb, and As are carcinogenic and toxic but only exist in very small mass concentrations (Table S3).

### Comparison of health risk potentials through inhalation of TSP- and PM_2.5_-bound metals

Inhalable dust fraction consists of particles with an aerodynamic diameter up to 100 µm (WHO 1999). Thus, the largest particles in TSP can be inhaled and deposited in the air passages between the mouth, the nose, and the larynx (WHO 1999; Wippich et al. 2020). Smaller particles with an aerodynamic diameter up to 10 µm in TSP are able to form respirable fraction and reach the gas-exchange region of the lungs (ISO 7708,1995; WHO 1999; Wippich et al. 2020). However, the EPA guidance used for health risk estimation in this study assumes that 100 percent of deposited dose is available for uptake into the systemic circulation or for activity in the respiratory tracts without clearance mechanisms of large particles (US EPA 2009). Hence, the largest particles in TSP may result in an overestimation of dose (i.e., C in Eq. (3)) in health risk estimation via inhalation. Recently, a method of interpreting TSP data was reported using a mathematical conversion ratio between inhalable and respirable particle depending on working activity and material including metal, fiber, and mineral (Wippich et al. 2020). Several studies have compared chemical compositions in different size of PM from specific emission sources (Li et al. 2017; Lyu et al. 2017; Tian et al. 2021) and however, the exact size distributions of airborne PM or metals are not yet available due to the variety of emission sources and physicochemical changes during the aging processes of particles (Li et al. 2017; Lyu et al. 2017; Tian et al. 2021).

Because metal mass concentrations in PM_10_ shown in Fig. 2 were not available from the real-time national monitoring stations (Korea Environmental Corporation 2021a), the mass concentrations of TSP-bound metals analyzed in this study were compared with those of PM_2.5_-bound metals in previous studies (Table S9). Several metals in TSP had lower values than those in PM_2.5_ probably due to differences in analysis method of metals and sampling date or locations. Seoul was considered more suitable for comparing the metal mass concentrations in TSP and PM_2.5_ samples because of nearer years of data collection (2013–2014) with those (2014–2015) in a previous study (Park et al 2020), and low variation in metal mass concentration between three sites in Seoul (Fig. 2; Table S3).

Additionally, carcinogenic and non-carcinogenic health risks by inhalation of TSP- bound 15 metals were estimated applying the ratios of metal mass concentrations in PM_2.5_ to those in TSP (Table S9; Table S10). For Ni and V that had higher mass concentrations in PM_2.5_ than in TSP, conversion ratio of 1 was used. For Cd and Co whose mass concentrations were not presented in the previous study (Park et al 2020), ratios were taken from a study conducted in Nanjing, China (Hu et al. 2012).

As a result of estimation of ILCR and HI in Seoul, cumulative ILCR and HI decreased by 23% and 29% respectively, ranging from 12 to 41% for each metal in ILCR and from 13 to 53% for each metal in HQ_i_ (Table S10). Similarly, a study on health risks associated with inhalation of metals in TSP and PM_2.5_ reported higher ILCR and HI values by inhalation of TSP-bound metals (As, Cd, Ni, and Pb) compared to by PM_2.5_-bound metals (As, Cd, Ni, and Pb), with a difference between 22 and 49% for each metal (Hu et al. 2012).

### Spatial and seasonal variability of health risks identified by ANOVA test

**Fig. 7:**
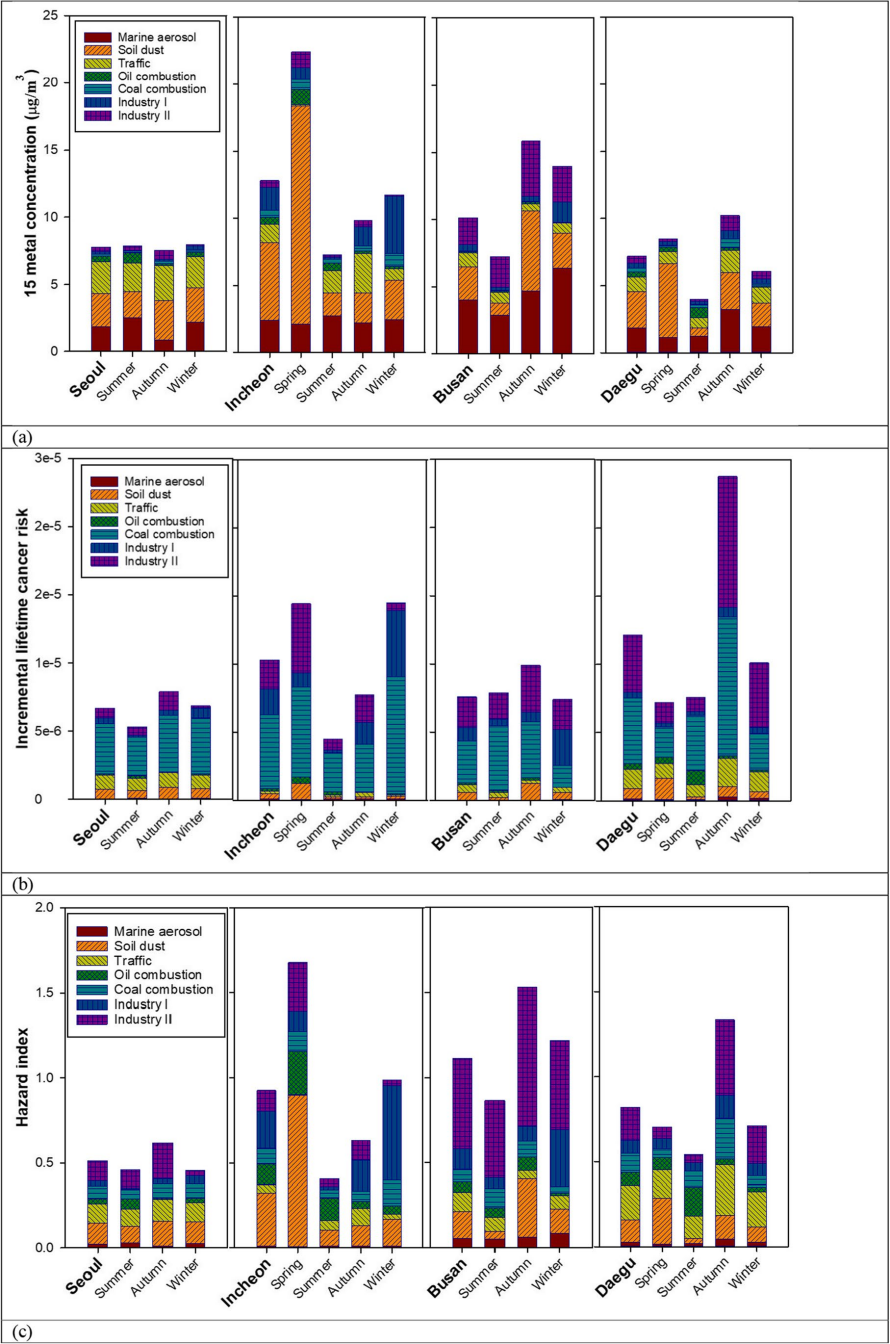
Metal concentration, ILCR, and HI values shown as contribution of sources in four cities by season: (a) contribution of sources to metal concentration; (b) contribution of sources to ILCR; (c) contribution of sources to HI

Figure 7 illustrates the seasonal variability of the mass concentrations of 15 metals, ILCR, and HI in the four cities. Unfortunately, TSP-bound metals were not sampled in spring in Seoul, and there were missing data for Pb, Ca, Mg, and Na in consecutive days in spring in Busan; thus, the ILCR and HI values in spring in both Seoul and Busan were not included in the seasonal variability analysis.

In Seoul, there was no statistically significant difference in ILCR and HI among summer, autumn, and winter, according to the ANOVA test. In Busan, significant differences in HI between summer and autumn were identified (*p* < 0.05). The lower HI in summer in Busan was due to the significantly different contribution of soil dust sources between summer and autumn (*p* < 0.001). Similarly, Incheon had the highest HI in spring, and the contribution of soil dust source to HI in spring was significantly different (*p* < 0.01) from in other seasons according to the ANOVA test.

ILCR was significantly higher (*p* < 0.05) in Incheon in spring and winter compared to autumn and summer. This seasonal variability in the ILCR of Incheon was mainly due to the significantly different contribution of the industry II source (*p* < 0.05) and industry I source (*p* < 0.001) in spring and winter, respectively, compared to those in both summer and autumn. In Daegu, ILCR and HI in autumn were higher compared to those in other seasons (*p* < 0.001 for ILCR, *p* < 0.01 for HI). This was mainly due to the larger contribution of industrial activities related sources (industry I, *p* < 0.01; industry II, *p* < 0.05) in autumn than in the other three seasons, the influence of a higher portion of coal combustion in autumn than in spring and winter (*p* < 0.05), and higher contribution of traffic in autumn than in summer (*p* < 0.05).

## Conclusion

In this study, airborne particulate matter-bound metals were analyzed at 14 sites in four metropolitan cities in South Korea between August 2013 and June 2017. The contribution of various emission sources to 15 metal mass concentrations and health risks were assessed using risk analysis coupled with source apportionment. The non-carcinogenic risks due to exposure to eight metals (Cd, Co, Ni, Pb, As, Al, Mn, and V) were greater than the HI of 1 at four sites located at or near the industrial complexes. Cumulative carcinogenic risk assessed by inhalation of five metals (Cd, Co, Ni, Pb, and As) exceeded the 10^−6^ cancer benchmark at all 14 sites and 10^−5^ at six sites, which includes four sites with HI greater than 1. To reduce the health risk potential due to inhalation of airborne metals, the following needs to be considered.

First, the management of anthropogenic sources (i.e., coal combustion and industrial activity-related sources) of toxic metals should be strengthened based on the risk-weighted contributions of sources. The largest contributors to carcinogenic risk due to inhalation of TSP-bound metals were coal combustion in three cities and industrial activity-related sources in one city. Industrial activity-related sources were the largest contributors to non-carcinogenic risk in three cities. Contributions of coal combustion sources to carcinogenic risks (39.4% to 55.1%) in the four cities were remarkably enhanced compared to the contributions to 15 metal mass concentration (from 0.18 to 4.64%). Similarly, the contributions of industrial sources to the metal mass concentrations ranged from 6.8 to 24.6%; however, the contribution of industrial sources to HI were distinctly higher, ranging from 28.6 to 58.8%.

Second, region-specific measures on the management of toxic metals should be established considering the major sources of metals contributing to the health risks of local people. While industrial sources were the largest contributor to HI in three of the four cities, soil dust contributed the most to HI in Incheon. Significantly different ILCR and HI values in Busan and Daegu were identified, respectively, and these were found to be due to the higher contribution of industry sources at a certain site in the respective city.

## Supplementary Information

Below is the link to the electronic supplementary material.

Supplementary file1 (DOCX 101 KB)

## Data Availability

Metal data analyzed during this study are included in supplementary information files. The data (i.e., PM_10_, NO_2_, and CO) that support the findings of this study are available from the Airkorea website (https://www.airkorea.or.kr/web/last_amb_hour_data?pMENU_NO=123). Hourly meteorological data that support the findings of this study can be obtained from automatic weather stations (https://data.kma.go.kr/data/grnd/selectAwsRltmList.do?pgmNo=56).
