# Supplementary material for: Sources of airborne particulate matter-bound metals and spatial-seasonal variability of health risk potentials in four large cities, South Korea
Source: Environ Sci Pollut Res Int. 2022 Jan 6;29(19):28359–74. doi: 10.1007/s11356-021-18445-8 (PMC8993791; doi:10.1007/s11356-021-18445-8)
Supplement: Supplementary file 1 — Supplementary file1 (DOCX 101 KB) [file 11356_2021_18445_MOESM1_ESM.docx]

Supplementary material

**Environmental Science and Pollution Research**

**Sources of airborne particulate matter-bound metals and spatial-seasonal variability of health risk potentials in four large cities, South Korea**

Eunhwa Choi^a^, Seung-Muk Yi^b^; Young Su Lee^c^; Hyeri Jo^c^; Sung-Ok Baek^d ;^ Jong-Bae Heo^e*^

^a^ [Institute of Construction and Environmental Engineering](http://icee.snu.ac.kr/), Seoul National University, 1 Gwanak-ro, Gwanak-gu, Seoul 08826, Republic of Korea

^b^ Department of Environmental Health Sciences, Graduate School of Public Health, Seoul National University, 1 Gwanak-ro, Gwanak-gu, Seoul, Republic of Korea

^c^ Department of Civil and Environmental Engineering, College of Engineering, Seoul National University, 1 Gwanak-ro, Gwanak-gu, Seoul 08826, Republic of Korea

^d^ Department of Environmental Engineering, Yeungnam University, Gyeongsan, 38541, Republic of Korea

^e^ Busan Development Institute, Busan 47210, Republic of Korea

**^*^** Corresponding author:

Jong-Bae Heo, Ph.D

E-mail: jbheo@bdi.re.kr

Phone: +82-51-860-8701, Fax: +82-51-860-8787

Table S1. Locations and classifications of study sites

Table S2. Method detection limit and mean recovery (%) of 15 metals analyzed by ICP/AES

Table S3. Mean concentration (±standard deviation) of TSP-bound 15 metals analyzed at 14 study sites

Table S4. Comparisons of source contributions to airborne TSP-bound 15 metal and PM_2.5_ mass concentrations

Table S5. Carcinogenic health risks estimated as cumulative ILCR (%) by source at 14 sites

Table S6. Non-carcinogenic health risk estimated as HI_s_ (%) at 14 sites.

Table S7. Pearson correlation coefficients between metal mass concentrations by source and ILCR/HI in four cities

Table S8. Pearson correlation coefficients between mass concentrations of TSP, PM_10_, metals and estimated ILCR/HI at 14 sites

Table S9. Comparison of mean mass concentrations of TSP- and PM_2.5_- bound metals

Table S10. Comparison of health risk potentials through inhalation of TSP- and PM_2.5_- bound metals

**Table S1. Locations and classifications of study sites**

| **Sampling sites** | **District classification** | **Location** |
| --- | --- | --- |
| **Seoul** |  |  |
| - GN (Seoul #1) | Residential/Roadside | 37°51'75"N 127°04'79"E |
| - GR (Seoul #2) | Residential/Roadside | 37°49'84"N 126°89'01'"E |
| - ST (Seoul #3) | Roadside | 37°55'59"N 126°97'23"E |
| **Incheon** |  |  |
| - GS (Incheon #1) | Residential/Roadside | 37°54'60"N 126°73'00"E |
| - GW (Incheon #2) | Near industrial complex | 37°44'96"N 126°72'40"E |
| - SE (Incheon #3) | Port area/near industrial complex | 37°46'38"N 126°64'98"E |
| - YH (Incheon #4) | Residential/Roadside | 37°54'54"N 126°67'59"E |
| **Busan** |  |  |
| - GA (Busan #1) | Residential/Near beach | 35°15'28"N 129°10'78"E |
| - SJ (Busan #2) | Port area | 35°12'95"N 129°04'54"E |
| - YS (Busan #3) | Residential area | 35°18'47"N 129°07'85"E |
| - HJ (Busan #4) | In industrial complex | 35°14'62"N 128°98'41"E |
| **Daegu** |  |  |
| - NW (Daegu #1) | In industrial complex/Roadside | 35°89'38"N 128°56'29"E |
| - DM (Daegu #2) | Near industrial complex | 35°84'69"N 128°57'04"E |
| - MC (Daegu #3) | Residential area | 35°86'59"N 128°63'98"E |

Table S2. Method detection limit and mean recovery (%) of 15 metals analyzed by ICP/AES

|  | **MDL (ng/m^3^)** | **Mean recovery (%)** |
| --- | --- | --- |
| **Al** | 10.32 | 56.5 |
| **Cd** | 0.05 | 89.3 |
| **Co** | 0.08 | 88.9 |
| **Fe** | 10.65 | 84.3 |
| **K** | 3.22 | 64.2 |
| **Mg** | 2.23 | 86.1 |
| **Mn** | 0.1 | 83.8 |
| **Na** | 10.93 | 58.2 |
| **Ni** | 0.1 | 96.3 |
| **Pb** | 0.3 | 108.5 |
| **Zn** | 1.48 | 80.6 |
| **Ca** | 8.78 | - |
| **Ti** | 1.32 | 45.6 |
| **V** | 0.06 | 80.3 |
| **As** | 0.24 | 91.3 |

**Table S3. Mean concentration (**±**standard deviation) of TSP-bound 15 metals analyzed at 14 study sites**

(unit: ng/m^3^) ^a^

| **Site** | | **TSP**  (µg/m^3^) | **PM_10_**  (µg/m^3^) | **Sum of 15 metals**  (µg/m^3^) | **Al** | **Cd** | **Co** | **Fe** | **K** | **Mg** | **Mn** | **Na** | **Ni** | **Pb** | **Zn** | **Ca** | **Ti** | **V** | **As** |
| --- | --- | --- | --- | --- | --- | --- | --- | --- | --- | --- | --- | --- | --- | --- | --- | --- | --- | --- | --- |
| **Seoul** | **Total N^b^** | 85 | - | - | 85 | 85 | 85 | 85 | 85 | 85 | 85 | 85 | 85 | 85 | 85 | 85 | 85 | 85 | 85 |
|  | **Mean (SD)** | 83.87  (31.81) | 34.74  (15.61) | 7.7 | 1,330  (732) | 1.22  (1.86) | 0.75  (0.38) | 1760  (882) | 505  (252.6) | 483  (218.7) | 41.1  (22.33) | 1590  (1052) | 5.4  (5.11) | 34.0  (24.07) | 137  (73.4) | 1750  (735) | 67  (31.9) | 4.23  (2.81) | 4.1  (3.54) |
|  | **#1 GN** | 82.38  (32.95) | 32.50  (15.42) | 7.4 | 1,170  (661) | 0.95  (1.23) | 0.62  (0.35) | 1,410  (650) | 463  (236.6) | 476  (189.3) | 35.0  (20.21) | 2,050  (985) | 5.6  (6.21) | 31.9  (23.68) | 122  (70.6) | 1,600  (576) | 56  (28.5) | 3.78  (2.63) | 3.5  (3.02) |
|  | **#2 GR** | 85.36  (31.11) | 33.05  (15.62) | 7.8 | 1,390  (797) | 1.34  (1.95) | 0.81  (0.44) | 1,500  (771) | 526  (286.8) | 541  (241.7) | 39.8  (23.38) | 1,850  (849) | 6.1  (5.70) | 36.1  (26.03) | 131  (76.5) | 1,670  (771) | 62  (31.6) | 4.82  (3.47) | 4.7  (4.12) |
|  | **#3 ST** | Not available | 38.67  (15.57) | 8.0 | 1,450  (725) | 1.37  (2.35) | 0.82  (0.33) | 2,500  (830) | 530  (229.8) | 422  (213) | 50.0  (21.49) | 740  (855) | 4.2  (1.87) | 34.1  (22.81) | 161  (69.4) | 2,010  (816) | 84  (30) | 4.07  (2.02) | 4.1  (3.39) |
| **Incheon** | **Total N^b^** | 112 | - | - | 112 | 112 | 112 | 112 | 112 | 112 | 112 | 104 | 112 | 112 | 112 | 112 | 112 | 112 | 112 |
|  | **Mean (SD)** | 117.26  (75.95) | 56.26  (36.47) | 12.8 | 2,650  (2626) | 1.34  (1.31) | 1.05  (1.17) | 2,370  (2030) | 1,048  (862.5) | 915  (810.2) | 76.2  (65.37) | 2790  (1408) | 10.2  (8.39) | 57.2  (47.50) | 96  (100.8) | 2,620  (1949) | 99  (87.3) | 12.24  (15.51) | 6.9  (6.20) |
|  | **#1 GS** | 122.30  (78.20) | 55.80  (32.04) | 11.4 | 2,490  (2,910) | 1.27  (1.28) | 1.14  (1.24) | 2,430  (2,344) | 1,005  (953.9) | 901  (960.4) | 72.6  (72.06) | 1,712  (1,089) | 8.9  (8.48) | 50.8  (36.51) | 98  (126.1) | 2,560  (2284) | 96  (99.3) | 10.10  (12.82) | 7.0  (6.66) |
|  | **#2 GW** | 130.71  (76.17) | 57.66  (43.25) | 14.4 | 2,800  (2,354) | 1.63  (1.57) | 1.10  (1.27) | 2,540  (1,918) | 1,140  (769.6) | 1,025  (728.9) | 77.9  (58.20) | 3,620  (1,094) | 10.2  (7.36) | 69.7  (72.18) | 107  (113.1) | 2,880  (1,744) | 104  (77.9) | 11.93  (14.19) | 8.0  (6.78) |
|  | **#3 SE** | 110.81  (70.27) | 65.08  (42.01) | 13.4 | 2,630  (2,411) | 1.24  (1.31) | 1.02  (1.01) | 2,300  (1,835) | 1,022  (837.0) | 872  (702.4) | 84.6  (67.43) | 3,400  (1,400) | 12.7  (8.91) | 60.3  (41.44) | 98  (92.0) | 2,830  (1,649) | 100  (76.8) | 16.25  (18.81) | 6.2  (5.53) |
|  | **#4 YH** | 105.23  (80.33) | 46.52  (24.99) | 11.8 | 2,660  (2,910) | 1.22  (1.06) | 0.93  (1.18) | 2,220  (2,084) | 1,024  (918.2) | 863  (856.7) | 69.6  (65.64) | 2,480  (1,184) | 8.9  (8.61) | 48.2  (26.78) | 84  (65.5) | 2,200  (2,081) | 94  (97.0) | 10.69  (15.68) | 6.4  (5.92) |
| **Busan** | **Total N^b^** | 158 | - | - | 156 | 158 | 158 | 158 | 157 | 135 | 158 | 127 | 158 | 128 | 158 | 140 | 158 | 158 | 158 |
|  | **Mean (SD)** | 91.46  (38.79) | 44.66  (18.66) | 10.5 | 1,170  (863) | 2.49  (5.64) | 0.98  (1.48) | 3,170  (4,501) | 471  (284.3) | 715  (389) | 107.1  (155.83) | 2,880  (1557) | 20.1  (31.88) | 49.7  (35.09) | 29  (50.4) | 1780  (1160) | 46  (32.3) | 7.87  (8.31) | 3.1  (4.74) |
|  | **#1 GA** | 82.29  (28.18) | 39.26  (14.51) | 7.5 | 830  (525) | 1.28  (1.05) | 0.18  (0.26) | 770  (483) | 377  (194) | 718  (382) | 30.4  (17.34) | 3,020  (1,331) | 4.0  (3.15) | 31.9  (26.85) | 5  (3) | 1,700  (998) | 27  (14.6) | 5.12  (5.12) | 2.5  (4.47) |
|  | **#2 SJ** | 89.15  (39.95) | 40.65  (16.54) | 8.3 | 1,130  (868) | 2.21  (2.44) | 0.46  (0.47) | 1,450  (969) | 485  (296.9) | 667  (394.7) | 52.6  (34.25) | 2,820  (1,623) | 9.8  (8.49) | 40.7  (24.51) | 8  (4.99) | 1,620  (1,176) | 43  (25.6) | 12.37  (13.00) | 3.1  (4.44) |
|  | **#3 YS** | 83.64  (39.31) | 44.42  (17.61) | 8.4 | 1,170  (766) | 1.49  (1.350) | 0.31  (0.35) | 1,250  (728) | 458  (267.4) | 697  (377) | 46.0  (26.60) | 2,950  (1,591) | 5.2  (3.57) | 35.3  (26.92) | 7  (5.7) | 1,750  (1,125) | 47  (25.5) | 5.99  (5.11) | 2.7  (5.46) |
|  | **#4 HJ** | 110.47  (40.74) | 55.90  (22.18) | 17.3 | 1,510  (1,074) | 4.95  (10.51) | 2.95  (1.74) | 9,090  (5,610) | 566  (337.3) | 777  (409.5) | 296.1  (215.17) | 2,710  (1,706) | 60.6  (41.47) | 80.1  (33.44) | 95  (64.9) | 2,060  (1,301) | 67  (43.4) | 8.04  (5.51) | 4.2  (4.48) |
| **Daegu** | **Total N^b^** | 83 | - | - | 83 | 83 | 83 | 83 | 83 | 83 | 83 | 83 | 83 | 83 | 83 | 83 | 83 | 83 | 83 |
|  | **Mean (SD)** | 99.24  (32.74) | 47.54  (15.96) | 7.2 | 1,330  (979) | 1.58  (1.07) | 2.65  (3.52) | 1,560  (849) | 484  (274.2) | 533  (308) | 51.8  (27.85) | 1,570  (962) | 13.1  (13.22) | 55.9  (83.92) | 15  (16.1) | 1,470  (847) | 50  (32.5) | 4.32  (2.34) | 4.7  (4.68) |
|  | **#1 NW** | 104.38  (34.08) | 53.91  (18.71) | 7.2 | 1,300  (918) | 1.80  (1.18) | 1.89  (1.31) | 1,860  (884) | 456  (259.3) | 512  (290.3) | 61.4  (30.57) | 1,500  (972) | 13.7  (6.85) | 45.0  (22.63) | 29  (21.3) | 1,400  (785) | 50  (30.5) | 4.35  (2.35) | 4.9  (5.26) |
|  | **#2 DM** | 100.20  (31.77) | 47.21  (14.67) | 7.4 | 1,390  (1,039) | 1.77  (1.16) | 4.91  (5.16) | 1,470  (818) | 523  (283.3) | 572  (334.2) | 52.5  (26.84) | 1,650  (1,058) | 19.9  (19.04) | 78.3  (139.66) | 8  (4.2) | 1,580  (871) | 50  (34) | 4.61  (2.67) | 4.7  (4.51) |
|  | **#3 MC** | 92.91  (32.49) | 41.43  (11.25) | 6.7 | 1,280  (1,008) | 1.17  (0.72) | 1.11  (1.04) | 1,350  (784) | 470  (285.2) | 515  (305.2) | 41.1  (22.57) | 1,540  (872) | 5.6  (4.20) | 44.0  (24.66) | 7  (3.5) | 1,420  (903) | 49  (34.1) | 4.00  (1.97) | 4.4  (5.01) |

^a^ ng/m^3^ was used as the unit if not specified.

^b^ A total of 15 elements were analyzed for each sample and however, some metals in the samples had missing values

**Table S4. Comparisons of source contributions to airborne TSP-bound 15 metal and PM_2.5_ mass concentrations**

(unit: %)

|  | **TSP** | | | | | | | | | | | | | | | | | | **PM_2.5_** | | |
| --- | --- | --- | --- | --- | --- | --- | --- | --- | --- | --- | --- | --- | --- | --- | --- | --- | --- | --- | --- | --- | --- |
|  | **This study** | | | | | | | | | | | | | | | | | | **Park et al (2020)** | **Choi et al (2013)** | **Jeong et al (2017)** |
| **Measurement year** | **2013-2014** | | | | **2014-2015** | | | | | **2015-2016** | | | | | **2016-2017** | | | | **2014-2015** | **2009-2010** | **2013** |
| **Site** | **Seoul** | | | | **Incheon** | | | | | **Busan** | | | | | **Daegu** | | | | **Seoul** | **Incheon** | **Busan** |
|  | **Mean** | **#1 GN** | **#2 GR** | **#3 ST** | **Mean** | **#1 GS** | **#2 GW** | **#3 SE** | **#4 YH** | **Mean** | **#1 GA** | **#2 SJ** | **#3 YS** | **#4 HJ** | **Mean** | **#1 NW** | **#2 DM** | **#3 MC** |  | **#3 SE** | **#3 YS** |
| **Marine aerosol** | 24.2 | 34.8 | 27.1 | 11.3 | 18.9 | 10.3 | 22.7 | 22.1 | 18.6 | 39.8 | 67.4 | 44.2 | 50.0 | 20.3 | 26.7 | 24.3 | 23.9 | 31.7 | 0.96 | 5.9 | - |
| **Soil dust** | 31.7 | 27.4 | 34.5 | 32.9 | 45.0 | 50.2 | 42.2 | 42.3 | 46.4 | 24.1 | 16.6 | 30.4 | 30.9 | 21.0 | 36.7 | 29.6 | 36.0 | 45.7 | 8.2 | 6.1 | 18 |
| **Traffic** | 30.0 | 24.6 | 21.9 | 43.1 | 10.6 | 14.0 | 9.31 | 10.3 | 9.29 | 10.4 | 10.4 | 14.4 | 12.5 | 7.33 | 15.6 | 31.8 | 7.46 | 6.74 | 23.3 | 23 | 18 |
| **Oil combustion** | 5.27 | 5.31 | 6.70 | 3.83 | 4.07 | 3.83 | 3.35 | 5.37 | 3.73 | 1.04 | 0.94 | 2.11 | 0.91 | 0.61 | 4.77 | 4.55 | 5.23 | 4.47 | 9.1 | - | 7 |
| **Coal combustion** | 2.08 | 1.80 | 2.49 | 1.93 | 4.64 | 5.68 | 4.66 | 3.88 | 4.48 | 0.18 | 0.20 | 0.22 | 0.19 | 0.14 | 4.25 | 4.48 | 4.10 | 4.21 | 4.2 | - | - |
| **Industry I** | 2.26 | 1.90 | 2.63 | 2.23 | 13.1 | 11.2 | 14.5 | 12.6 | 13.8 | 4.68 | 3.21 | 4.96 | 3.38 | 5.84 | 5.06 | 3.00 | 6.92 | 5.16 | - | 8.5 | 3 |
| **Industry II** | 4.58 | 4.20 | 4.76 | 4.75 | 3.81 | 4.91 | 3.31 | 3.50 | 3.72 | 19.9 | 1.33 | 3.76 | 2.22 | 44.9 | 7.01 | 2.17 | 16.4 | 2.03 |  |  |  |
| **Sum of source contribution** | 100 | 100 | 100 | 100 | 100 | 100 | 100 | 100 | 100 | 100 | 100 | 100 | 100 | 100 | 100 | 100 | 100 | 100 | 100 **^a^** | 100 **^b^** | 100 **^c^** |
| **15 metal concentration** (µg/m^3^) | 7.7 | 7.4 | 7.8 | 8.0 | 12.8 | 11.4 | 14.4 | 13.4 | 11.8 | 10.5 | 7.5 | 8.3 | 8.4 | 17.3 | 7.2 | 7.2 | 7.4 | 6.7 | - | - | - |
| **TSP concentration** (µg/m^3^) | 83.87 | 82.38 | 85.36 | n.a | 117.26 | 122.3 | 130.71 | 110.81 | 105.23 | 91.46 | 82.29 | 89.15 | 83.64 | 110.47 | 99.24 | 104.38 | 100.2 | 92.91 | - | - | - |
| **PM_2.5_ concentration** (µg/m^3^) |  |  |  |  |  |  |  |  |  |  |  |  |  |  |  |  |  |  | 42.6 | 42.6 | 26 |

^a^ Other sources extracted by PMF: Secondary nitrate (19.0%); Secondary sulfate (20.1%); Biomass burning (12.2%); Roadway (3.03%)

^b^ Other sources extracted by PMF: Secondary nitrate (25.4%); Secondary sulfate (19.0%); Combustion (6.1%); Biomass burning (6.1%)

^c^ Other sources extracted by PMF: Secondary nitrate (19%); Secondary sulfate (31%); Road dust: 1.04 (4%)

**Table S5. Carcinogenic health risks estimated as cumulative ILCR** **(%) by source at 14 sites**

| **Source** | **Seoul** | | | | **Incheon** | | | | | **Busan** | | | | | **Daegu** | | | |
| --- | --- | --- | --- | --- | --- | --- | --- | --- | --- | --- | --- | --- | --- | --- | --- | --- | --- | --- |
|  | **Average** | **#1 GN** | **#2 GR** | **#3 ST** | **Average** | **#1 GS** | **#2 GW** | **#3 SE** | **#4 YH** | **Average** | **#1 GA** | **#2 SJ** | **#3 YS** | **#4 HJ** | **Average** | **#1 NW** | **#2 DM** | **#3 MC** |
| **Marine aerosol** | 5.39E-08  (0.81) | 7.45E-08 (1.34) | 6.15E-08 (0.83) | 2.58E-08 (0.37) | 1.07E-07 (1.04) | 5.13E-08 (0.49) | 1.46E-07 (1.27) | 1.32E-07 (1.37) | 9.95E-08 (1.05) | 2.98E-08 (0.39) | 3.58E-08 (0.9) | 2.61E-08 (0.46) | 2.95E-08 (0.63) | 2.41E-08 (0.16) | 1.59E-07 (1.32) | 1.49E-07(1.39) | 1.49E-07 (0.85) | 1.80E-07 (2.25) |
| **Soil dust** | 7.07E-07  (10.6) | 5.87E-07 (10.5) | 7.83E-07 (10.5) | 7.51E-07 (10.7) | 4.08E-07 (3.96) | 3.99E-07 (3.83) | 4.34E-07 (3.76) | 4.02E-07 (4.15) | 3.97E-07 (4.16) | 5.66E-07 (7.46) | 2.85E-07 (7.11) | 5.81E-07 (10.2) | 5.90E-07 (12.5) | 8.08E-07 (5.07) | 7.46E-07 (6.13) | 6.06E-07(5.65) | 7.5E-07 (4.28) | 8.71E-07 (10.9) |
| **Traffic** | 9.92E-07  (14.8) | 7.80E-07 (14.0) | 7.35E-07 (9.83) | 1.46E-06 (20.8) | 1.55E-07 (1.51) | 1.79E-07 (1.72) | 1.55E-07 (1.35) | 1.59E-07 (1.65) | 1.28E-07 (1.34) | 5.49E-07 (7.24) | 4.03E-07 (10.1) | 6.19E-07 (10.9) | 5.37E-07 (11.4) | 6.36E-07 (4.0) | 1.38E-06 (11.4) | 2.88E-06(26.8) | 6.92E-07 (3.91) | 5.67E-07 (7.06) |
| **Oil combustion** | 1.03E-07  (1.54) | 9.93E-08 (1.79) | 1.33E-07 (1.78) | 7.66E-08 (1.1) | 2.10E-07 (2.04) | 1.72E-07 (1.65) | 1.95E-07 (1.69) | 2.90E-07 (3.0) | 1.81E-07 (1.9) | 1.43E-07 (1.89) | 9.57E-08 (2.39) | 2.38E-07 (4.2) | 1.02E-07 (2.16) | 1.39E-07 (0.88) | 4.55E-07 (3.74) | 4.41E-07  (4.11) | 5.20E-07 (2.94) | 4.04E-07 (5.02) |
| **Coal combustion** | 3.69E-06  (55.1) | 3.05E-06 (54.8) | 4.50E-06 (60.2) | 3.50E-06 (49.9) | 5.40E-06 (52.4) | 5.79E-06 (55.6) | 6.15E-06 (53.2) | 4.74E-06 (48.9) | 4.91E-06 (51.3) | 3.08E-06 (40.6) | 2.59E-06 (64.6) | 3.02E-06 (53.2) | 2.69E-06 (56.9) | 4.01E-06 (25.2) | 4.79E-06 (39.4) | 5.10E-06 (47.5) | 4.79E-06 (27.1) | 4.46E-06 (55.5) |
| **Industry I** | 4.19E-07  (6.26) | 3.37E-07 (6.06) | 4.96E-07 (6.63) | 4.23E-07 (6.04) | 1.92E-06 (18.6) | 1.43E-06 (13.7) | 2.40E-06 (20.8) | 1.93E-06 (19.9) | 1.90E-06 (19.9) | 9.81E-07 (12.9) | 4.93E-07 (12.3) | 8.47E-07 (14.9) | 5.77E-07 (12.2) | 2.01E-06 (12.6) | 4.18E-07 (3.44) | 2.52E-07 (2.35) | 5.98E-07 (3.38) | 4.04E-07 (5.03) |
| **Industry II** | 7.31E-07  (10.9) | 6.42E-07 (11.5) | 7.73E-07 (10.3) | 7.77E-07 (11.1) | 2.11E-06 (20.5) | 2.39E-06 (23.0) | 2.08E-06 (18) | 2.04E-06 (21.1) | 1.95E-06 (20.4) | 2.24E-06 (29.5) | 1.10E-07 (2.75) | 3.46E-07 (6.1) | 2.04E-07 (4.32) | 8.31E-06 (52.2) | 4.22E-06 (34.7) | 1.31E-06 (12.2) | 1.02E-05 (57.6) | 1.15E-06 (14.3) |
| **Sum** | 6.69E-06  (100) | 5.57E-06 (100) | 7.48E-06 (100) | 7.01E-06 (100) | **1.03E-05 (100)** | **1.04E-05**  **(100)** | **1.16E-05 (100)** | 9.69E-06 (100) | 9.57E-06 (100) | 7.59E-06 (100) | 4.01E-06 (100) | 5.68E-06 (100) | 4.73E-06 (100) | **1.59E-05 (100)** | **1.22E-05 (100)** | **1.07E-05 (100)** | **1.77E-05 (100)** | 8.03E-06 (100) |

**Table S6. Non-carcinogenic health risk estimated as HI_s_ (%) at 14 sites.**

| **Source** | **Seoul** | | | | **Incheon** | | | | | **Busan** | | | | | **Daegu** | | | |
| --- | --- | --- | --- | --- | --- | --- | --- | --- | --- | --- | --- | --- | --- | --- | --- | --- | --- | --- |
|  | **Average** | **#1 GN** | **#2 GR** | **#3 ST** | **Average** | **#1 GS** | **#2 GW** | **#3 SE** | **#4 YH** | **Average** | **#1 GA** | **#2 SJ** | **#3 YS** | **#4 HJ** | **Average** | **#1 NW** | **#2 DM** | **#3 MC** |
| **Marine aerosol** | 2.19E-02 (4.29) | 3.03E-02  (6.87) | 2.50E-02 (4.67) | 1.05E-02 (1.88) | 9.24E-03 (1.00) | 4.42E-03 (0.51) | 1.26E-02 (1.26) | 1.14E-02 (1.19) | 9.24E-03 (1.06) | 5.31E-02 (4.77) | 6.58E-02 (15.7) | 4.80E-02 (6.86) | 5.42E-02 (9.7) | 4.43E-02 (1.6) | 2.70E-02 (3.28) | 2.51E-02 (2.94) | 2.53E-02 (2.42) | 3.05E-02 (5.36) |
| **Soil dust** | 1.25E-01 (24.4) | 1.04E-01 (23.5) | 1.38E-01 (25.8) | 1.33E-01 (23.7) | 3.16E-01 (34.1) | 3.09E-01 (35.6) | 3.36E-01 (33.3) | 3.11E-01 (32.6) | 3.07E-01 (35.2) | 1.58E-01 (14.1) | 7.95E-02 (18.9) | 1.62E-01 (23.1) | 1.64E-01 (29.3) | 2.25E-01 (8.1) | 1.31E-01 (16.0) | 1.07E-01 (12.5) | 1.34E-01 (12.7) | 1.54E-01 (27.0) |
| **Traffic** | 1.14E-01 (22.3) | 8.95E-02 (20.3) | 8.45E-02 (15.8) | 1.68E-01 (30.1) | 4.79E-02 (5.18) | 5.53E-02 (6.37) | 4.78E-02 (4.74) | 4.90E-02 (5.13) | 3.96E-02 (4.54) | 1.17E-01 (10.5) | 8.55E-02 (20.4) | 1.32E-01 (18.8) | 1.14E-01 (20.4) | 1.35E-01 (4.87) | 2.01E-01 (24.5) | 4.20E-01 (49.1) | 1.01E-01 (9.66) | 8.29E-02 (14.6) |
| **Oil combustion** | 3.10E-02 (6.06) | 2.99E-02 (6.78) | 4.00E-02 (7.46) | 2.31E-02 (4.14) | 1.19E-01 (12.8) | 9.77E-02 (11.3) | 1.11E-01 (11.0) | 1.64E-01 (17.2) | 1.02E-01 (11.8) | 5.89E-02 (5.29) | 3.93E-02 (9.36) | 9.76E-02 (14.0) | 4.18E-02 (7.48) | 5.69E-02 (2.05) | 7.58E-02 (9.21) | 7.36E-02 (8.61) | 8.67E-02 (8.28) | 6.72E-02 (11.8) |
| **Coal combustion** | 7.39E-02 (14.5) | 6.13E-02 (13.9) | 9.03E-02 (16.8) | 7.03E-02 (12.6) | 9.61E-02 (10.4) | 1.03E-01 (11.9) | 1.09E-01 (10.9) | 8.43E-02 (8.82) | 8.74E-02 (10.0) | 7.34E-02 (6.59) | 6.17E-02 (14.7) | 7.19E-02 (10.3) | 6.42E-02 (11.5) | 9.57E-02 (3.45) | 1.14E-01 (13.8) | 1.21E-01 (14.2) | 1.14E-01 (10.9) | 1.06E-01 (18.6) |
| **Industry I** | 3.01E-02 (5.89) | 2.43E-02 (5.51) | 3.57E-02 (6.66) | 3.04E-02 (5.46) | 2.18E-01 (23.6) | 1.63E-01 (18.8) | 2.74E-01 (27.2) | 2.20E-01 (23.0) | 2.16E-01 (24.8) | 1.24E-01 (11.1) | 6.23E-02 (14.8) | 1.07E-01 (15.3) | 7.29E-02 (13.0) | 2.54E-01 (9.13) | 7.79E-02 (9.46) | 4.70E-02 (5.5) | 1.11E-01 (10.6) | 7.53E-02 (13.3) |
| **Industry II** | 1.16E-01 (22.7) | 1.02E-01 (23.2) | 1.23E-01 (22.9) | 1.23E-01 (22.1) | 1.20E-01 (13.0) | 1.35E-01 (15.6) | 1.18E-01 (11.7) | 1.16E-01 (12.1) | 1.10E-01 (12.7) | 5.31E-01 (47.7) | 2.60E-02 (6.21) | 8.20E-01 (11.7) | 4.84E-02 (8.65) | 1.97 (70.9) | 1.97E-01 (23.9) | 6.12E-02 (7.16) | 4.76E-01 (45.4) | 5.35E-02 (9.41) |
| **Sum** | 0.51 (100) | 0.44 (100) | 0.54 (100) | 0.56 (100) | 0.93 (100) | 0.87 (100) | **1.01 (100)** | 0.96 (100) | 0.87 (100) | **1.11 (100)** | 0.42 (100) | 0.7 (100) | 0.56 (100) | **2.78 (100)** | 0.82 (100) | 0.86 (100) | **1.05 (100)** | 0.57 (100) |

**Table S7. Pearson correlation coefficients between metal mass concentrations by source and ILCR/HI in four cities**

| **Sources** | **Seoul** | | | | **Incheon** | | | | **Busan** | | | | **Daegu** | | | |
| --- | --- | --- | --- | --- | --- | --- | --- | --- | --- | --- | --- | --- | --- | --- | --- | --- |
|  | **ILCR** | | **HI** | | **ILCR** | | **HI** | | **ILCR** | | **HI** | | **ILCR** | | **HI** | |
|  | **Pearson's *r*** | **p-value** | **Pearson's *r*** | **p-value** | **Pearson's *r*** | **p-value** | **Pearson's *r*** | **p-value** | **Pearson's *r*** | **p-value** | **Pearson's *r*** | **p-value** | **Pearson's *r*** | **p-value** | **Pearson's *r*** | **p-value** |
| **Marine aerosol** | -.284^**^ | .007 | -.332^**^ | .001 | .018 | .851 | -.022 | .815 | .079 | .322 | .011 | .889 | .356^**^ | .001 | .231^*^ | .034 |
| **Soil dust** | .308^**^ | .003 | **.**654^***^ | .000 | .286^**^ | .002 | .**858^***^** | .000 | .359^*^**^*^**^*^ | .000 | .471^***^ | .000 | -.083 | .453 | .163 | .139 |
| **Traffic** | .126 | .237 | .490**^*^**^**^ | .000 | -.130 | .172 | -.181 | .056 | -.062 | .438 | -.010 | .901 | .139 | .206 | .414^*^**^*^**^*^ | .000 |
| **Oil combustion** | -.074 | .490 | .022 | .835 | .317^**^ | .001 | **.739^***^** | .000 | .170^*^ | .032 | .177^*^ | .025 | -.233^*^ | .033 | -.215^*^ | .050 |
| **Coal combustion** | **.912^***^** | .000 | .342^**^ | .001 | **.877^***^** | .000 | .240^*^ | .011 | .**718^***^** | .000 | .262^**^ | .001 | .639^***^ | .000 | .381^*^**^*^**^*^ | .000 |
| **Industry I** | .253^*^ | .016 | .309^**^ | .003 | .490^***^ | .000 | .265^**^ | .005 | .518^*^**^*^**^*^ | .000 | .457^*^**^*^**^*^ | .000 | .465**^*^**^**^ | .000 | .498^***^ | .000 |
| **Industry II** | .316^**^ | .002 | **.732^***^** | .000 | .402^*^**^*^**^*^ | .000 | **.795^***^** | .000 | **.729^***^** | .000 | **.958**^*^**^*^**^*^ | .000 | **.859^***^** | .000 | **.824^***^** | .000 |

Pearson correlation coefficient (two-tailed) *p* value: **p*< .05; ***p*< .01; ****p*< .001.

**Table S8. Pearson correlation coefficients between mass concentrations of TSP, PM_10_, metals and estimated ILCR/HI at 14 sites**

| **Concentration** |  | **Seoul** | | | | | | | | **Incheon** | | | | | | | | | |
| --- | --- | --- | --- | --- | --- | --- | --- | --- | --- | --- | --- | --- | --- | --- | --- | --- | --- | --- | --- |
|  |  | **Seoul** | | **#1 GN** | | **#2 GR** | | **#3 ST** | | **Incheon** | | **#1 GS** | | **#2 GW** | | **#3 SE** | | **#4 YH** | |
|  |  | ILCR | HI | ILCR | HI | ILCR | HI | ILCR | HI | ILCR | HI | ILCR | HI | ILCR | HI | ILCR | HI | ILCR | HI |
| TSP | Pearson's r | .628^***^ | .896^***^ | .537^**^ | .806^***^ | .657^***^ | .903^***^ | na | na | .624^***^ | .988^***^ | .488^**^ | .962^***^ | .609^**^ | .981^***^ | .662^***^ | .949^***^ | .642^***^ | .973^***^ |
|  | p-value | .000 | .000 | .002 | .000 | .000 | .000 | na | na | .000 | .000 | .008 | .000 | .001 | .000 | .000 | .000 | .000 | .000 |
| PM10 | Pearson's r | .522^**^ | .864^***^ | .588^**^ | .852^***^ | .533^**^ | .875^***^ | .238 | .772^***^ | .739^***^ | .975^***^ | .621^***^ | .949^***^ | .680^***^ | .969^***^ | .775^***^ | .971^***^ | .748^***^ | .916^***^ |
|  | p-value | .003 | .000 | .001 | .000 | .002 | .000 | .205 | .000 | .000 | .000 | .000 | .000 | .000 | .000 | .000 | .000 | .000 | .000 |
| 15 metals | Pearson's r | .284 | .739^***^ | .394^*^ | .724^***^ | .366^*^ | .783^***^ | -.054 | .670^***^ | .405^*^ | .929^***^ | .384^*^ | .951^***^ | .402^*^ | .933^***^ | .407^*^ | .895^***^ | .558^**^ | .968^***^ |
|  | p-value | .128 | .000 | .031 | .000 | .047 | .000 | .798 | .000 | .033 | .000 | .044 | .000 | .034 | .000 | .032 | .000 | .002 | .000 |
| 8 metals | Pearson's r | .371^*^ | .761^***^ | .451^*^ | .798^***^ | .444^*^ | .769^***^ | .086 | .700^***^ | .408^*^ | .930^***^ | .279 | .908^***^ | .396^*^ | .918^***^ | .444^*^ | .919^***^ | .479^**^ | .937^***^ |
|  | p-value | .043 | .000 | .012 | .000 | .014 | .000 | .683 | .000 | .031 | .000 | .151 | .000 | .037 | .000 | .018 | .000 | .010 | .000 |
| 5 metals ^b^ | Pearson's r | .654^***^ | .885^***^ | .672^***^ | .858^***^ | .604^***^ | .899^***^ | .572^**^ | .872^***^ | .781^***^ | .753^***^ | .750^***^ | .704^***^ | .695^***^ | .748^***^ | .657^***^ | .619^***^ | .774^***^ | .761^***^ |
|  | p-value | .000 | .000 | .000 | .000 | .000 | .000 | .003 | .000 | .000 | .000 | .000 | .000 | .000 | .000 | .000 | .000 | .000 | .000 |
| **Concentration** |  | **Busan** | | | | | | | | | | **Daegu** | | | | | | | |
|  |  | **Busan** | | **#1 GA** | | **#2 SJ** | | **#3 YS** | | **#4 HJ** | | **Daegu** | | **#1 NW** | | **#2 DM** | | **#3 MC** | |
|  |  | ILCR | HI | ILCR | HI | ILCR | HI | ILCR | HI | ILCR | HI | ILCR | HI | ILCR | HI | ILCR | HI | ILCR | HI |
| TSP | Pearson's r | .411^**^ | .518^**^ | .480^**^ | .704^***^ | .433^**^ | .760^***^ | .156 | .472^**^ | .621^***^ | .668^***^ | .341 | .613^**^ | .463^*^ | .734^***^ | .262 | .411^*^ | .272 | .653^**^ |
|  | p-value | .008 | .001 | .005 | .000 | .006 | .000 | .335 | .002 | .000 | .000 | .076 | .001 | .013 | .000 | .178 | .030 | .169 | .000 |
| PM10 | Pearson's r | .564^***^ | .578^***^ | -.093 | .267 | .646^***^ | .709^***^ | .398^*^ | .786^***^ | .546^**^ | .506^**^ | .466^*^ | .762^***^ | .358 | .853^***^ | .430 | .638^**^ | .542^**^ | .866^***^ |
|  | p-value | .000 | .000 | .618 | .146 | .000 | .000 | .012 | .000 | .002 | .004 | .012 | .000 | .062 | .000 | .052 | .002 | .003 | .000 |
| 15 metals | Pearson's r | .426^*^ | .776^***^ | .190 | .572^**^ | .276 | .735^***^ | .107 | .584^**^ | .731^***^ | .845^***^ | .503^**^ | .757^***^ | .449^*^ | .780^***^ | .538^**^ | .654^***^ | .416^*^ | .806^***^ |
|  | p-value | .019 | .000 | .324 | .001 | .148 | .000 | .573 | .001 | .000 | .000 | .006 | .000 | .016 | .000 | .003 | .000 | .031 | .000 |
| 8 metals ^a^ | Pearson's r | .340 | .765^***^ | .133 | .582^**^ | .161 | .704^***^ | .136 | .663^***^ | .469^**^ | .672^***^ | .192 | .525^**^ | .187 | .604^**^ | .237 | .411^*^ | .175 | .652^***^ |
|  | p-value | .066 | .000 | .492 | .001 | .404 | .000 | .472 | .000 | .009 | .000 | .327 | .004 | .340 | .001 | .224 | .030 | .382 | .000 |
| 5 metals ^b^ | Pearson's r | .908^***^ | .621^***^ | .860^***^ | .773^***^ | .865^***^ | .737^***^ | .902^***^ | .767^***^ | .882^***^ | .833^***^ | .600^***^ | .617^***^ | .697^***^ | .821^***^ | .542^**^ | .526^**^ | .862^***^ | .722^***^ |
|  | p-value | .000 | .000 | .000 | .000 | .000 | .000 | .000 | .000 | .000 | .000 | .001 | .000 | .000 | .000 | .003 | .004 | .000 | .000 |

Pearson correlation coefficient (two-tailed) *p* value: **p*< .05; ***p*< .01; ****p*< .001.

na denotes not available

^a^ Cd, Co, Ni, Pb, As, Al, Mn, and V

^b^ Cd, Co, Ni, Pb, and As

**Table S9. Comparison of mean mass concentrations of TSP- and PM_2.5_- bound metals**

(unit: ng/m^3^) ^a^

|  | **TSP** | | | | **PM_2.5_** | | | **TSP** | **PM_2.5_** | **Conversion ratio calculated in this study** | |
| --- | --- | --- | --- | --- | --- | --- | --- | --- | --- | --- | --- |
| **Source** | **This study** | | | | **Park et al (2020)** | **Choi et al (2013)** | **Jeong et al (2017)** | **Hu et al (2012)** | **Hu et al (2012)** | **This study; Park et al (2020)** | **Hu et al (2012)** |
| Measurement year | 2013-2014 | 2014-2015 | 2015-2016 | 2016-2017 | 2014-2015 | 2009-2010 | 2013 | 2010 | 2010 | 2013-2014; 2014-2015 | 2010 |
| Site | Seoul  average | Incheon  #3 SE | Busan  #3 YS | Daegu  average | Seoul | Incheon  #3 SE | Busan  #3 YS | Nanjing, China | Nanjing, China | Seoul, Korea | Nanjing, China |
| TSP  concentration (µg/m^3^) | 83.87 | 110.81 | 83.64 | 99.24 |  |  |  | - | - |  |  |
| PM_2.5_ concentration (µg/m^3^) |  |  |  |  | 42.6 | 42.6 | 26 |  |  |  |  |
| Al | 1,330 | 2,630 | 1,170 | 1,330 | 628 | 177.5 |  | - | - | **0.47** | - |
| Cd | 1.22 | 1.24 | 1.49 | 1.58 | - | 2.1 | 3.1 | 4.2 | 3.0 | - | **0.71** |
| Co | 0.75 | 1.02 | 0.31 | 2.65 | - | 0.8 | 0.8 | 7.2 | 6.3 | - | **0.88** |
| Mn | 41.1 | 84.6 | 46.0 | 51.8 | 27 | 35.8 | 25.0 | 146.8 | 80.9 | **0.66** | 0.55 |
| Ni | 5.4 | 12.7 | 5.2 | 13.1 | 91 | 18.8 | - | 28.4 | 14.1 | - | 0.50 |
| Pb | 34.0 | 60.3 | 35.3 | 55.9 | 20 | 57.6 | 30 | 235.9 | 180.3 | **0.59** | 0.76 |
| V | 4.23 | 16.25 | 5.99 | 4.32 | 10 | 73.9 | 8.3 | - | - | - | - |
| As | 4.1 | 6.2 | 2.7 | 4.7 | 3 | 100.1 | 5.7 | 14.0 | 9.9 | **0.73** | 0.71 |

^a^ ng/m^3^ was used as the unit if not specified.

**Table S10. Comparison of health risk potentials through inhalation of TSP- and PM_2.5_- bound metals**

| **Metal** | **ILCR** | | **HQ*_i_*** | | **Conversion ratio used in this study** | **Source** |
| --- | --- | --- | --- | --- | --- | --- |
|  | **TSP based** | **Converted to**  **PM_2.5_ based** | **TSP based** | **Converted to**  **PM_2.5_ based** |  |  |
| **As** | 4.21E-06 | 3.08E-06 | 0.065 | 0.048 | 0.73 | Park et al (2020), This study |
| **Co** | 1.62E-06 | 1.43E-06 | 0.030 | 0.026 | 0.88 | Hu et al (2012) |
| **Ni** | 2.62E-07 | 2.62E-07 | 0.078 | 0.078 | 1 | This study |
| **Cd** | 5.11E-07 | 3.63E-07 | 0.028 | 0.020 | 0.71 | Hu et al (2012) |
| **Pb** | 9.71E-08 | 5.73E-08 | 0.054 | 0.032 | 0.59 | Park et al (2020), This study |
| **Al** |  |  | 0.064 | 0.030 | 0.47 | Park et al (2020), This study |
| **V** |  |  | 0.010 | 0.010 | 1 | This study |
| **Mn** |  |  | 0.200 | 0.132 | 0.66 | Park et al (2020), This study |
| **Cumulative ILCR** | 6.70E-06 | 5.18E-06 |  |  |  |  |
| **HI** |  |  | 0.529 | 0.376 |  |  |
| **Difference (%)** |  | 23 |  | 29 |  |  |

**References**

1. Choi J, Heo J-B, Soo-Jin B, et al (2013) Source apportionment of PM2.5 at the coastal area in Korea. Science of the Total Environment 447:370–380
2. Hu X, Zhang Y, Ding Z, et al (2012) Bioaccessibility and health risk of arsenic and heavy metals (Cd, Co, Cr, Cu, Ni, Pb, Zn and Mn) in TSP and PM2.5 in Nanjing, China. Atmos Environ 57:146–152
3. Jeong J-H, Shon Z-H, Kang M, et al (2017) Comparison of source apportionment of PM2.5 using receptor models in the main hub port city of East Asia: Busan. Atmospheric Environment 148:115–127. <https://doi.org/10.1016/j.atmosenv.2016.10.055>
4. Park EH, Heo J, Kim H, Yi S-M (2020) Long term trends of chemical constituents and source contributions of PM2.5 in Seoul. Chemosphere 251:126371
